# The potential value of the use of berberine in depression: a systematic review and meta-analysis of preclinical studies

**DOI:** 10.3389/fphar.2025.1664784

**Published:** 2025-11-03

**Authors:** Bifang Yao, Zhengxiang Long, Xiaojiao Lin, Guangqiang Chen, Xiaoyu Li, Ziqi Ye, Jiahong Liu

**Affiliations:** ^1^ The Affiliated Kangning Hospital of Wenzhou Medical University, Zhejiang Provincial Clinical Research Center for Mental Health, Wenzhou, China; ^2^ Wenzhou Kangning Hospital Group, Wenzhou, China

**Keywords:** depression, berberine, preclinical, mechanisms, meta-analysis

## Abstract

**Background:**

Depression is a prevalent global disorder that imposes a significant burden on individuals worldwide. Berberine is a promising candidate for future antidepressant therapies; however, no comprehensive systematic evaluation has been conducted to date.

**Methods:**

Five electronic databases—PubMed, Embase, Web of Science, OVID, and the Cochrane Library—were systematically searched to identify preclinical studies investigating the antidepressant effects of berberine. Outcomes were assessed using the standardized mean difference with 95% confidence intervals to evaluate overall effect sizes. Study quality was evaluated using the 10-item Systematic Review Centre for Laboratory Animal Experimentation risk of bias tool. Publication bias was assessed if more than 10 studies were included in an analysis.

**Results:**

A total of 20 preclinical studies evaluating berberine‘s antidepressant effects were identified. Berberine administration was associated with reduced depression-like behaviors. Specifically, Berberine significantly: increased body weight (n = 7; SMD = 1.67; 95% CI: 0.57 to 2.76; *P* < 0.00001),Reduced immobility time in the tail suspension test (n = 9; SMD = −2.41; 95% CI: −3.15 to −1.67; *P* = 0.01),Increased sucrose consumption (n = 12; SMD = 1.82; 95% CI: 1.29 to 2.34; *P* = 0.02),Reduced immobility time in the forced swim test (n = 17; SMD = −2.35; 95% CI: −2.91 to −1.79; *P* < 0.00001),Increased total movement distance in the open field test (n = 7; SMD = 1.70; 95% CI: 0.58 to 2.81; *P* < 0.00001),Increased time spent in the open field test (n = 3; SMD = 1.02; 95% CI: 0.44 to 1.60; *P* = 0.92), Increased the number of crossings in the open field test (n = 4; SMD = 0.76; 95% CI: 0.20 to 1.33; *P* = 0.23). Furthermore, berberine was found to reduce levels of inflammatory markers, enhance neurotransmitter levels (excluding dopamine), and elevate brain-derived neurotrophic factor levels.

**Conclusion:**

Berberine consistently demonstrated antidepressant-like effects in preclinical models and showed preliminary potential mechanisms of action. However, the limitations of current studies highlight the necessity for more comprehensive preclinical research and well-designed clinical trials.

## 1 Introduction

Depression is a widespread and formidable mental health affliction that impacts individuals worldwide. Between 1990 and 2019, the number of incident cases of depression increased by 49.86% (Liu et al., 2019). As of 2019, depression ranked among the top three causes of disability-adjusted life years among females and was the 13th leading cause of disability-adjusted life years across all age groups in 204 countries (GBD, 2019 Diseases and Injuries Collaborators, 2020). This condition imposes significant public health challenges and places a heavy burden on families. Depression is characterized by a high likelihood of recurrence throughout the lifespan (Assoc, 2013), can occur at any age (Alexopoulos, 2005; Donohue et al., 2019), and presents with a heterogeneous symptom profile (Fried and Nesse, 2015; Fried et al., 2014). To date, the underlying pathological and pharmacological mechanisms of depression remain complex and poorly understood. Various factors have been implicated in its onset and progression, including immune dysregulation (Bai et al., 2024; Bullmore, 2018; Drevets et al., 2022), monoamine imbalance (Malhi and Mann, 2018), age-specific neurofunctional changes (Bore et al., 2024), and gut microbiota metabolism (Aburto and Cryan, 2024; Zhao et al., 2024).

Currently, first-line treatments for depression include antidepressant medications and psychological therapies (Simon et al., 2024). In recent years, novel treatments targeting neurotransmitter systems have garnered increasing interest (De Risio et al., 2020; Njenga et al., 2024; Tang et al., 2025). However, depression remains a largely incurable condition, particularly in cases of treatment-resistant depression. The heterogeneity of depressive symptoms poses a major barrier to effective treatment (Fried, 2017), and a substantial proportion of patients fail to achieve meaningful improvement with existing therapies (Cuijpers et al., 2020). Moreover, the initiation of antidepressant medications is often associated with adverse effects, including weight changes (Gill et al., 2020), sexual dysfunction (Peleg et al., 2022), gastrointestinal disturbances (Oliva et al., 2021), and an increased risk of suicidality (Hetrick et al., 2021; Boaden et al., 2020).

Due to the limited efficacy of current treatments and the occurrence of serious adverse effects, there is an urgent need to identify innovative therapeutic approaches to combat depression. Recently, increasing attention has been directed toward berberine (BBR), an isoquinoline alkaloid with potential therapeutic benefits (Shayganfard, 2023). BBR is a bioactive compound isolated from medicinal herbs and has traditionally been used in the treatment of gastrointestinal disorders (Kong et al., 2004; Kulkarni and Dhir, 2010; Dong et al., 2022). Over the past 2 decades, BBR has demonstrated a wide range of pharmacological activities across various disease domains, including diabetes (Wang et al., 2024; Xie et al., 2022), cancer (Hsu et al., 2024; Sajeev et al., 2024; Yan et al., 2024), Parkinson’s disease (Wang et al., 2021), and cardiovascular disorders (Zhao et al., 2021). Given the complex multifactorial pathology of depression, BBR emerges as a promising therapeutic candidate due to its multiple pharmacological actions, including anti-inflammatory, antioxidant, and neuroprotective effects (Imanshahidi and Hosseinzadeh, 2008; Wang et al., 2017). Its multi-target mode of action is expected to overcome the limitations of conventional single-target drugs.

Recent *in vivo* and *in vitro* studies have provided positive therapeutic evidence suggesting that BBR holds significant potential for the treatment of depression (Chen and Zhang, 2025). Notably, previous research has demonstrated that BBR can enhance the effects of conventional antidepressants (Kulkarni and Dhir, 2008), primarily by modulating neurotransmitter levels and their associated receptor systems. BBR exerts its antidepressant effects through multiple pharmacological mechanisms. These include inhibition of the NLRP3 inflammasome (Qin et al., 2023), upregulation of brain-derived neurotrophic factor (BDNF) expression (Zhan et al., 2021), and improvement of hypothalamic-pituitary-adrenal axis function (Gao et al., 2024). After crossing the blood–brain barrier, BBR can enhance hippocampal neurogenesis (Yang et al., 2023) and exert neuroprotective effects (Wang et al., 2005; Yoo et al., 2006).

Taken together, these findings indicate that BBR may represent a novel, multimodal antidepressant that operates through mechanisms distinct from those of traditional antidepressant medications. Despite BBR’s diverse pharmacological and biochemical activities, its precise mechanisms of action remain unclear. Notably, no meta-analysis has yet been performed to synthesize and summarize the role of BBR in depression based on preclinical studies. To address this gap and enhance our understanding of BBR’s synergistic effects and underlying molecular mechanisms in depression, we systematically reviewed preclinical studies using animal models. This review endeavors to establish a robust and comprehensive body of evidence in support of future clinical investigations into the antidepressant properties of BBR.

## 2 Methods

The current systematic review and meta-analysis was designed and conducted in accordance with the Preferred Reporting Items for Systematic Reviews and Meta-Analyses (PRISMA) guidelines (Vrabel, 2009; Shamseer et al., 2015). The study protocol, based on SYRCLE’s systematic review programme format for animal intervention studies (De Vries et al., 2015), was submitted to the INPLASY platform on 9 June 2025, and officially registered on 9 June 2025, under registration number INPLASY 202560037 (DOI:10.37766/inplasy2025.6.0037).

### 2.1 Literature search

Five online electronic databases—PubMed, OVID, Web of Science, Embase, and the Cochrane Library—were searched to obtain information on animal studies investigating the use of BBR for depression. Two separate searches were conducted on 31 March 2025, by two independent reviewers (Ling XJ and Chen GQ), once in the morning and once in the afternoon. To minimize the possible of omitting relevant studies, the reference lists of all retrieved studies were manually screened. The search strategy employed a predefined set of MeSH terms and keywords applied to the full text. These terms included both disease-related and compound-related keywords, such as “depress*,” “sadness,” “berberine,” and “huangliansu.” (The complete search strategies are shown in the Table 1).

**TABLE 1:** The complete search strategies on Five electronic databases

Database	Step	Search query	Outcome
Embase	#1	Berbericase OR huangliansu OR “umbellatine”/exp OR umbellatine OR xiaopijian OR barberry OR “berberis”/exp OR “berberis” OR “berberine”/exp OR berberine	15693
	#2	[“depression”/exp OR “depression” OR “sadness”/exp OR “sadness” OR “melancholia”/exp OR “melancholia” OR “suicide”/exp OR “suicide” OR “dysthymia”/exp OR “dysthymia” OR “major depression”/exp OR “major depression” OR (major AND (“depression”/exp OR depression))] AND depressive	211812
	#3	#1 AND #2	61
PubMed	#1	((((Berber*) OR (huangliansu)) OR (barberry)) OR (Berberine)) OR (xiaopijian)	14,254
	#2	[(((((((depress*) OR (Sadness)) OR (Melancholia*)) OR (suicide)) OR (dysthymi*)) OR (depression)) OR (depressive)) OR (depressive symptom*)] OR (depressive disorders)	810826
	#3	(#1) AND (#2)	230
Web of Science	#1	[((TS=(Berber*)) OR TS=(huanglianshu)) OR TS=(umbellamine)] OR TS=(xiaolijian)	29655
	#2	[(((TS=(depress*)) OR TS=(Sadness)) OR TS=(Melancholia*)) OR TS=(suicide)] OR TS=(dysthymi*)	1806254
	#3	(#1) AND (#2)	432
Cochrane Library	#1	(Berber*):ti,ab,kw OR (huanglianshu):ti,ab,kw OR (umbellamine):ti,ab,kw OR (xiaolijian):ti,ab,kw	554
	#2	(depress*):ti,ab,kw OR (Sadness):ti,ab,kw OR (Melancholia*):ti,ab,kw OR (suicide):ti,ab,kw OR (dysthymi*):ti,ab,kw	129980
	#3	#1 AND #2	11
OVID	#1	(Berber* or huangliansu or Umbellatine or xiaopijian).af	19261
	#2	(depress* or Sadness or Melancholia* or suicide or dysthymi*).af	1324213
	#3	#1 AND #2	284

### 2.2 Inclusion and exclusion criteria

After removing duplicates, two different reviewers (Ling XJ and Chen GQ) independently screened each article based on the PICOS criteria without mutual consultation. Any discrepancies were resolved by consulting a third independent reviewer (Long ZX).

#### 2.2.1 Inclusion criteria

1) Results of studies published as an original article. 2) The subjects must be animals and there are no restrictions on the method of construction of the animal model, gender, size, species or sample size. 4) Studies with separate BBR treatment and control or model groups were available. 5) Outcome measures associated with depression -like behaviors 6) No restriction on the language.

#### 2.2.2 Exclusion criteria

1) Reviews, patents, clinical studies, case reports, conference and book chapter. 2) No full-text articles 3) Repeatedly published literature. 4) Experimental findings in the articles were incomplete. 5) Outcome measures were unqualified. 6) Preclinical studies that were inconsistencies in the study purpose.

### 2.3 Data extraction

After an initial review of the titles and abstracts of all studies and the exclusion of duplicates, full-text articles eligible for qualitative data extraction were summarized, tabulated, and independently assessed by two reviewers (Chen GQ and Li XY). For studies reporting experimental data at multiple time points, only the data from the final time point were extracted in our analysis. A meta-analysis was performed after the collection at least 3 studies per group. Finally, the data include: 1) The first author of the articles and the year of publication. 2) The species, sex, weight range, and sample size of the subjective animals. 3) The modeling method of the animal model of depression. 4) The dose, duration of BBR treatment. 5) Method of vehicle or BBR administration 6) Medication for control variables in the control or model group, dose and duration of drugs used. 7) 15 of outcome indicators: Weight, Sucrose preference in sucrose preference test (SPT), The number of crossings in OFT (Open field test), Total distance of movement in OFT, Time duration of center square in OFT, Immobility time in FST (Forced swim test), Immobility time in TST (Tail suspension test), Interleukin 6 (IL-6) levels, Interleukin-1β (IL-1β) levels, Tumor necrosis factor α (TNF-α) levels, 5-hydroxytryptamine (5-HT) levels, Norepinephrine (NE) levels, dopamine (DA) levels; Brain-derived neurotrophic factor (BDNF) protein levels, BDNF mRNA levels. all of data in article were obtained from the tables or graphs by Engauge Digitizer software. All included data were presented as mean ± standard deviation (SD). If the original outcomes in the articles were reported as the standard error of the mean (SEM), they were converted to SD using the formula: SD = SEM * √n (Lee et al., 2015).

### 2.4 Quality evaluation

To assess the quality of the included studies, two reviewers (Chen GQ and Li XY) independently evaluated the risk of bias using the 10-item SYRCLE risk of bias tool developed by the Center for the Evaluation of Laboratory Animal Experiments (Hooijmans et al., 2014). The tool assesses the following domains: selection bias (sequence generation, allocation concealment, random housing), performance bias, detection bias (random outcome assessment, blinding), attrition bias, reporting bias, and other sources of bias. Each item was rated as “low risk,” “high risk,” or “unclear risk.” Any discrepancies during the quality assessment process were resolved through consultation with a third reviewer (Yao BF) to reach a consensus.

### 2.5 Statistical analysis

Statistical analyses were conducted using Review Manager (RevMan) version 5.4.1 and STATA version 15.1. As the outcome indicators were continuous variables, results were evaluated using standardized mean differences (SMDs) and their corresponding 95% confidence intervals (CIs) to estimate the overall effect size.

Due to variations among the included studies in terms of species, age, sample size, dosage or administration of BBR, and experimental duration, a random-effects model was employed. In line with recent proposals to address the replication crisis (Benjamin et al., 2017), we employed a stricter significance threshold of p < 0.005. This *a priori* decision was made to reduce the likelihood of false positives and to report only the most robust effects. Heterogeneity was assessed using the I^2^ statistic. However, following the updated Cochrane Handbook, I^2^ values were no longer used as the sole criterion for selecting the effects model. The general interpretation of I^2^ was shown in Table 2.

**TABLE 2:** The meanings of I^2^.

I^2^	Meanings
0%–40%	May represent no serious heterogeneity
30%–60%	May represent moderate heterogeneity
50%–90%	May represent substantial heterogeneity
75%–100%	May represent considerable heterogeneity

When ten or more studies reported the same outcome indicators, Begg’s test and Egger’s test were used to assess potential publication bias. Sensitivity analysis was conducted by sequentially excluding each individual study to evaluate the robustness of the overall findings and identify any potentially influential studies.

## 3 Results

### 3.1 Study selection

**FIGURE 1:**
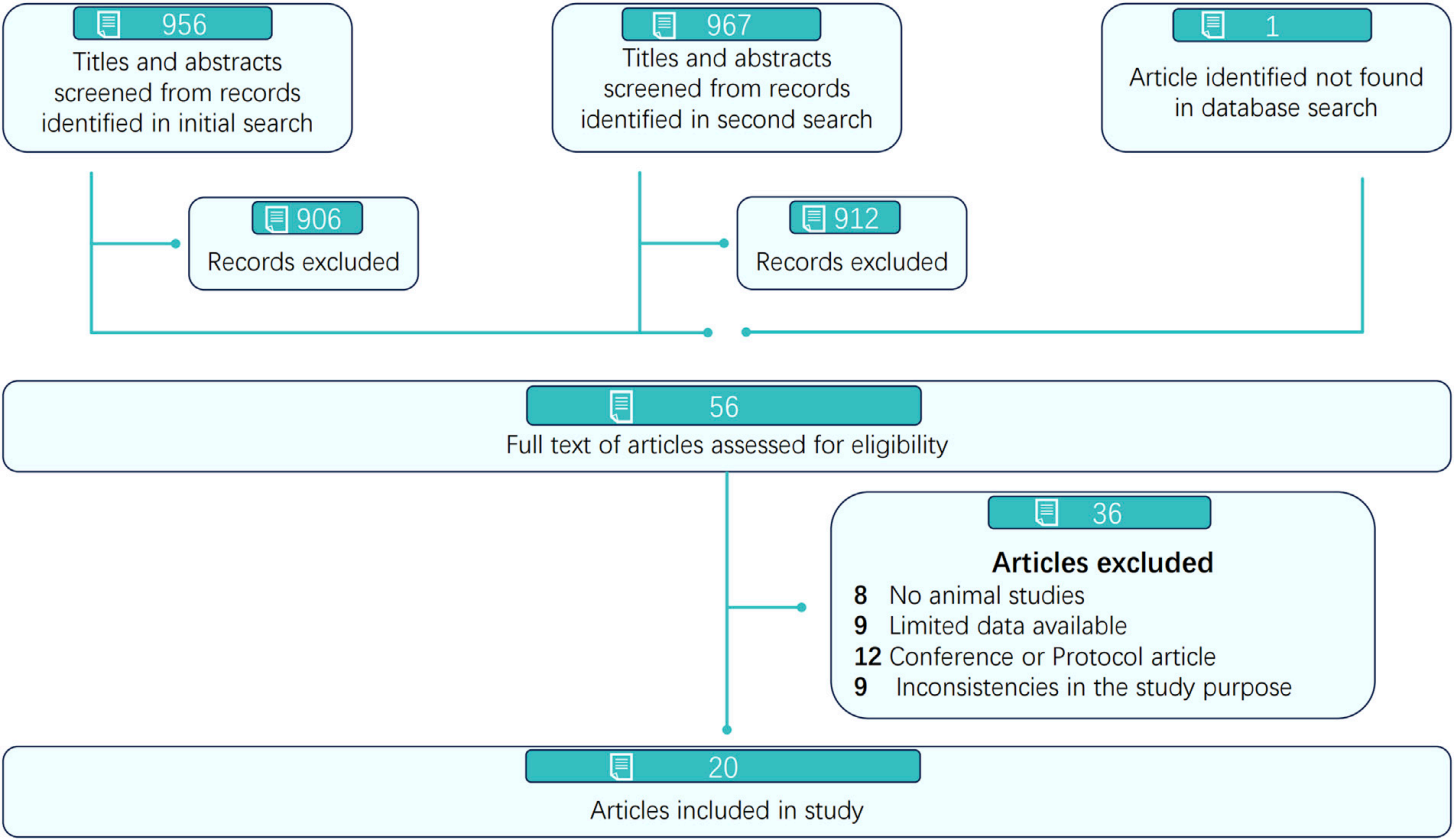
Prisma flow diagram of study selection and inclusion.

A total of 956 and 967 articles were identified from five electronic databases (PubMed, Embase, Web of Science, OVID, and the Cochrane Library) through two independent searches conducted on the same day at different times by two reviewers. After removing 359 and 362 duplicate articles, 597 and 605 articles remained and were screened by title and abstract by two reviewers (Ling XJ and Chen GQ), as detailed in Figure 1. Subsequently, 547 and 550 articles were excluded by each reviewer, respectively, resulting in 56 articles assessed for full-text eligibility. Ultimately, 20 articles published between 2007 and 2024 were included for methodological quality assessment and further analysis.

### 3.2 Article characteristics

Across the 20 included studies, two species of laboratory animals were used: rats (n = 5) and mice (n = 15). Specifically, 7 mouse and rat strains were reported: CD1 mice (n = 1), C57BL/6 mice (n = 5), ICR mice (n = 7), SD rats (n = 4), KM mice (n = 1), Wistar rats (n = 1), and albino mice (n = 2). One study involved two different mouse strains. Experimental and control groups consisted of 6–18 animals per group. The reported ages of the animals ranged from 4 weeks (approximately 1 month) to 12 weeks (approximately 3 months). However, seven studies did not report the age of the animals, and two studies made only vague references to the animals being adults. Male animals were used exclusively in 19 studies, while only one study included both male and female subjects. Reported body weights varied considerably across studies, primarily due to the differences in species and strains used. The details are shown in the Table 3.

**TABLE 3:** The characteristics of articles.

No.	Study	Year	Subjective					Model/control		BBR group			Model/control group			Outcome index
			Species	Age	Sex	n = BBR/model group	Weight	Method	Time	Administration	Drug dose	Duration	Administration	Drug dose	Duration	
1	Deng et al.	2018	CD1mice/C57/BL6j mice	8–9 weeks age/8 weeks	Male and female/male	8/8	No mention	CSDS (Chronic social defeat stress procedure)	10 days	Drinking	25,50 and 100 mg/kg/day	10 days	No mention			①②⑤⑥⑦⑧⑨⑩
2	Fan et al.	2017	ICR mice	No mention	Male	8/8	18–22 g	CORT injection (40 mg/kg)	21 days	Oral gavage	50 and 100 mg/kg/day	21 days	Oral gavage	Same volume of physiological saline	21 days	③⑦⑩
3	Gao et al.	2019	KM mice (昆明老鼠)	No mention	male	10/10	22–25 g	Lipopolysaccharide injection (0. 83 mg/kg)	once time	Gavage	25,50 and 100 mg/kg/day	7 days	Gavage	Same volume of physiological saline	7 days	②⑦⑭⑯
4	Gao et al.	2018	Sprague Dawley (SD) rat	8–10 weeks	Male	8/8	200–250 g	CUMS (Chronic unpredictable mild stress model)	5 weeks	Oral route	25,50 and 100 mg/kg/day	21 days	No mention			①②⑦⑧
5	Ge et al.	2023	C57BL/6 mice	No mention	Male	10/10	18–22 g	CUMS (Chronic unpredictable mild stress model)	4 weeks	Intragastrically	2.5,5,10 mg/kg/day	7 days	Intragastrically	0.5% CMC - Na solution	7 days	⑤⑥⑦⑫⑬⑭⑯
6	Huang et al.	2023	Wistar rats	No mention	Male	10/10	170 ± 10 g	CUMS (Chronic unpredictable mild stress model)	21 days	Intragastrically	50 and 100 mg/kg/day	14 days	Intragastrically	Saline (1 mL/100 g)	14 days	①②④⑤⑩⑪⑫⑬
7	Kulkarni et al.	2007	Albino mice (Laca strain)	No mention	Male	10/10	22–30 g	No mention		Intraperitoneally	2, 5, 10 and 20 mg/kg	No mention	No mention			⑦⑧
8	Kulkarni et al.	2008	Albino mice (Laca strain)	No mention	Male	10/10	22–30 g	No mention		Intraperitoneally	2, 5, 10 and 20 mg/kg/day	15 days	Intraperitoneally	Saline	15 days	⑦⑧⑪⑬
9	Lee et al.	2012	SD rats	Adult	male	6/6	260–280 g	Morphine injection (dose ranging from 10 to 50 mg/kg-body weigh) twice a day	10 days	No mention	10, 20 and 50 mg/kg	No mention	No mention	Saline	No mention	⑦
10	Liu et al.	2017	ICR mice	6 weeks	Male	10/10	22 ± 2 g	CUMS (Chronic unpredictable mild stress model)	4 weeks	Oral route	50 and 100 mg/kg/day	4 weeks	Oral route	0.9% saline containing 0.3% carboxymethyl cellulose	4 weeks	②⑭⑮⑯
11	Lu et al.	2021	ICR mice	4–6 weeks	Male	8/8	18–22 g	CUMS (Chronic unpredictable mild stress model)	4 weeks	No mention	50,100 and 200 mg/kg/day	4 weeks	No mention	0.9% sodium choloride solution	4 weeks	②⑦
12	Peng et al.	2017	ICR albino mice	3 months	Male	10/10	around 25 g	No mention		oral route	10, 20 and 100 mg/kg	No mention	Oral route	Saline(10 mL/kg body weight)	No mention	⑦⑧
13	Qin et al.	2023	SD rats	Adult	Male	18/18	260–280 g	CORT intragastrically (20 mg/kg/day)	35 days	Intragastrically	100 and 200 mg/kg/day	35 days	Intragastrically	CORT intragastrically (20 mg/kg/day)	35 days	②③⑤⑥⑦⑧⑭⑮⑯
14	Shen et al.	2016	ICR mice	No mention	Male	8/8	18–22 g	CORT injection	21 days	Oral gavage	50 and 100 mg/kg/day	21 days	Oral gavage	Physiological saline	21 days	①②③④⑦⑨⑩
15	Tang et al.	2024	C57BL/6 mice	7 weeks	Male	6/6	No mention	Chronic restraint stress	No mention	Oral gavage	200 and 300 mg/kg/day	21 days	Oral gavage	0.9% saline (10 mL/kg/day)	21 days	①⑤⑦⑧⑨⑩⑪⑫⑭⑮⑯
16	Wang et al.	2022	ICR mice	6 weeks	Male	10/10	22–24 g	CUMS (Chronic unpredictable mild stress model)	21 days	Oral route	25,50 and 100 mg/kg/day	21 days	Oral route	0.9% saline containing 0.3% carboxymethyl cellulose	21 days	①②③④⑤⑦⑧⑫
17	Xu et al.	2018	ICR mice	2 months	Male	10/10	25–30 g	The chronic inflammatory pain	14 days	Intraperitoneal injection	50 mg/kg/day	7 days	Intraplantar injection	Some volume of saline	7 days	⑦
18	Yang et al.	2023	C57BL/6J mice	7 weeks	Male	9/9	No mention	CUMS (Chronic unpredictable mild stress model)	28 days	Gavage	5 and 10 mg/kg/day	3 weeks	Gavage	Some volumes of distilled water	3 weeks	②⑤⑦⑧⑭⑯
19	Yi et al.	2021	C57BL/6J mice	8 weeks	Male	8/8	20–22 g	Chronic stress procedure	4 weeks	Oral route	100 mg/kg/day	4 weeks	Oral route	Saline	4 weeks	②
20	Zhu et al.	2017	SD rats	2 months	Male	10/10	200–220 g	CUMS (Chronic unpredictable mild stress model)	No mention	No mention	40 and 200 mg/kg/day	No mention	No mention	Some volume of 0.9% saline	No mention	1 ②⑦

①Weight ②Sucerose Prefence test③OFT The number of crossings ④OFT The number of rearings ⑤OFT Total distance⑥OFT Time duration of center square ⑦Immobility time in FST ⑧Immobility time in TST ⑨BDNF mRNA ⑩BDNF protein ⑪NE ⑫5-HT ⑬DA ⑭TNF-α ⑮IL-6 ⑯IL-1β.

### 3.3 Risk of bias

The SYRCLE risk of bias assessments for all included studies are summarized in Supplementary Table S4. Most studies demonstrated either a low risk or an unclear risk in the domains of sequence generation and baseline characteristics. With the exception of the study by Kulkarni and Dhir (2007), all others exhibited either unclear or high risk in these domains. Regarding random housing, all studies showed either unclear or low risk, except for the study by Yang L et al., which presented a higher risk. All studies showed good control in the domain of selective outcome reporting. For other domains, the risk of bias varied among individual studies. The details are shown in the Table 4.

**TABLE 4:** Risk of bias.

No.	Study	Year	Sequence generation (randomization)	Baseline characteristics	Allocation concealment	Random housing	Blinding (performance/Detection bias)	Random outcome assessment	Blinding	Incomplete outcome data	Selective outcome reporting	Other scources of bias
1	Deng et al.	2018	Unclear risk	Low risk	High risk	Low risk	Unclear risk	Unclear risk	Unclear risk	Unclear risk	Low risk	Low risk
2	Fan et al.	2017	Unclear risk	Low risk	High risk	Low risk	Unclear risk	Unclear risk	Low risk	Low risk	Low risk	Low risk
3	Gao et al.	2019	Unclear risk	Low risk	High risk	Low risk	High risk	Low risk	High risk	Low risk	Low risk	High risk
4	Gao et al.	2018	Low risk	Low risk	High risk	Low risk	High risk	Low risk	High risk	Low risk	Low risk	High risk
5	Ge et al.	2023	Unclear risk	Unclear risk	Unclear risk	Unclear risk	Unclear risk	Unclear risk	Unclear risk	Unclear risk	Low rrisk	Unclear risk
6	Huang et al.	2023	Unclear risk	Unclear risk	Unclear risk	Unclear risk	Unclear risk	Unclear risk	Unclear risk	Unclear risk	Low risk	Unclear risk
7	Kulkarni et al.	2007	Low risk	Low risk	Low risk	Unclear risk	Low risk	Low risk	Unclear risk	Low risk	Low risk	Low risk
8	Kulkarni et al.	2008	Low risk	Low risk	Unclear risk	Low risk	Low risk	Low risk	Unclear risk	Low risk	Low risk	Low risk
9	Lee et al.	2012	Unclear risk	Low risk	High risk	Unclear risk	Low risk	Unclear risk	Low risk	High risk	Low risk	Unclear risk
10	Liu et al.	2017	Unclear risk	Low risk	High risk	Unclear risk	High risk	High risk	High risk	High risk	Low risk	Unclear risk
11	Lu et al.	2021	Unclear risk	Unclear risk	High risk	Low risk	High risk	High risk	High risk	High risk	Low risk	Unclear risk
12	Peng et al.	2017	Unclear risk	Unclear risk	High risk	Low risk	High risk	High risk	High risk	High risk	Low risk	Unclear risk
13	Qin et al.	2023	Low risk	Low risk	High risk	Low risk	High risk	Low risk	Low risk	Low risk	Low risk	Low risk
14	Shen et al.	2016	Low risk	Low risk	Unclear risk	Low risk	High risk	Low risk	High risk	Low risk	Low risk	Low risk
15	Tang et al.	2024	Unclear risk	Unclear risk	Unclear risk	Unclear risk	High risk	Low risk	High risk	Low risk	Low risk	Low risk
16	Wang et al.	2022	Low risk	Low risk	Unclear risk	Low risk	High risk	Low risk	High risk	Low risk	Low risk	Low risk
17	Xu et al.	2018	Low risk	Low risk	Unclear risk	Unclear risk	High risk	Unclear risk	High risk	Low risk	Low risk	Low risk
18	Yang et al.	2023	Low risk	Low risk	High risk	High risk	High risk	High risk	High risk	Low risk	Low risk	Low risk
19	Yi et al.	2021	Low risk	Low risk	High risk	Low risk	High risk	Low risk	High risk	Low risk	Low risk	Unclear risk
20	Zhu et al.	2017	Unclear risk	Low risk	High risk	Low risk	High risk	Unclear risk	High risk	Low risk	Low risk	Unclear risk

### 3.4 Meta-analysis results

#### 3.4.1 Depression-like behaviors

This analysis included seven behavioral indicators related to depression-like symptoms reported across the 20 included studies: body weight, sucrose preference in the SPT, number of crossings in the OFT, total distance moved in the OFT, time spent in the center square in the OFT, immobility time in the FST, and immobility time in the TST.

**FIGURE 2:**
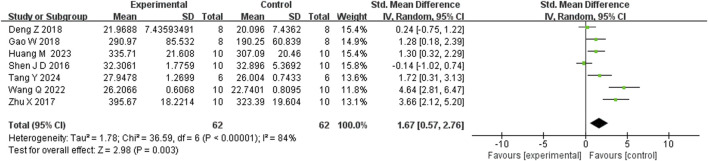
Forest plot: random effects meta-analysis of weight.

**FIGURE 3:**
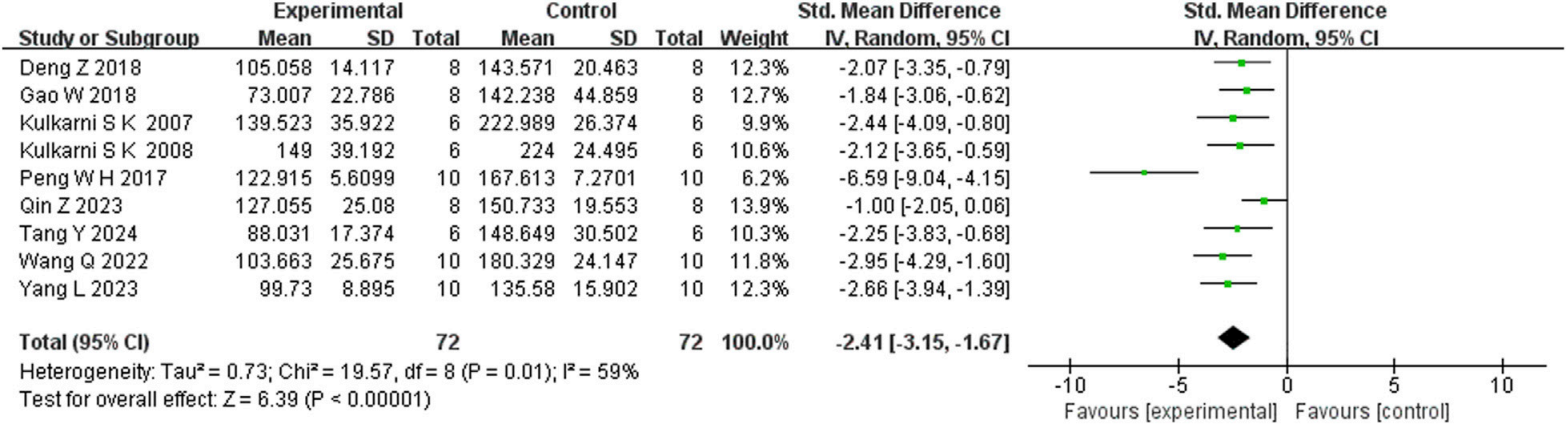
Forest plot: random effects meta-analysis of Immobility time in TST.

**FIGURE 4:**
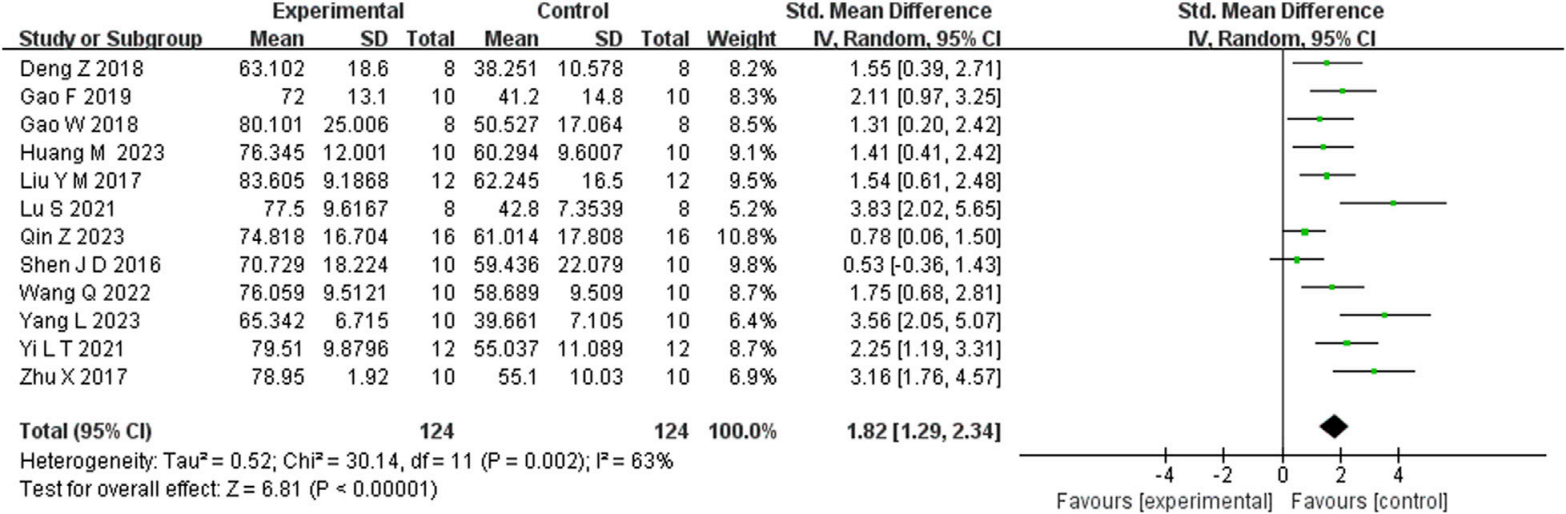
Forest plot: random effects meta-analysis of SPT.

Seven studies reported that BBR significantly increased body weight compared to controls (n = 7; SMD = 1.67; 95% CI: 0.57 to 2.76; heterogeneity: I^2^ = 84%, *P* < 0.00001; Figure 2). Nine studies showed that BBR reduced immobility time in the TST (n = 9; SMD = −2.41; 95% CI: 3.15 to −1.67; I^2^ = 59%, *P* = 0.01; Figure 3). Twelve studies demonstrated that BBR significantly increased sucrose preference (n = 12; SMD = −1.82; 95% CI: 2.34 to −1.29; I^2^ = 63%, *P* = 0.02; Figure 4).

**FIGURE 5:**
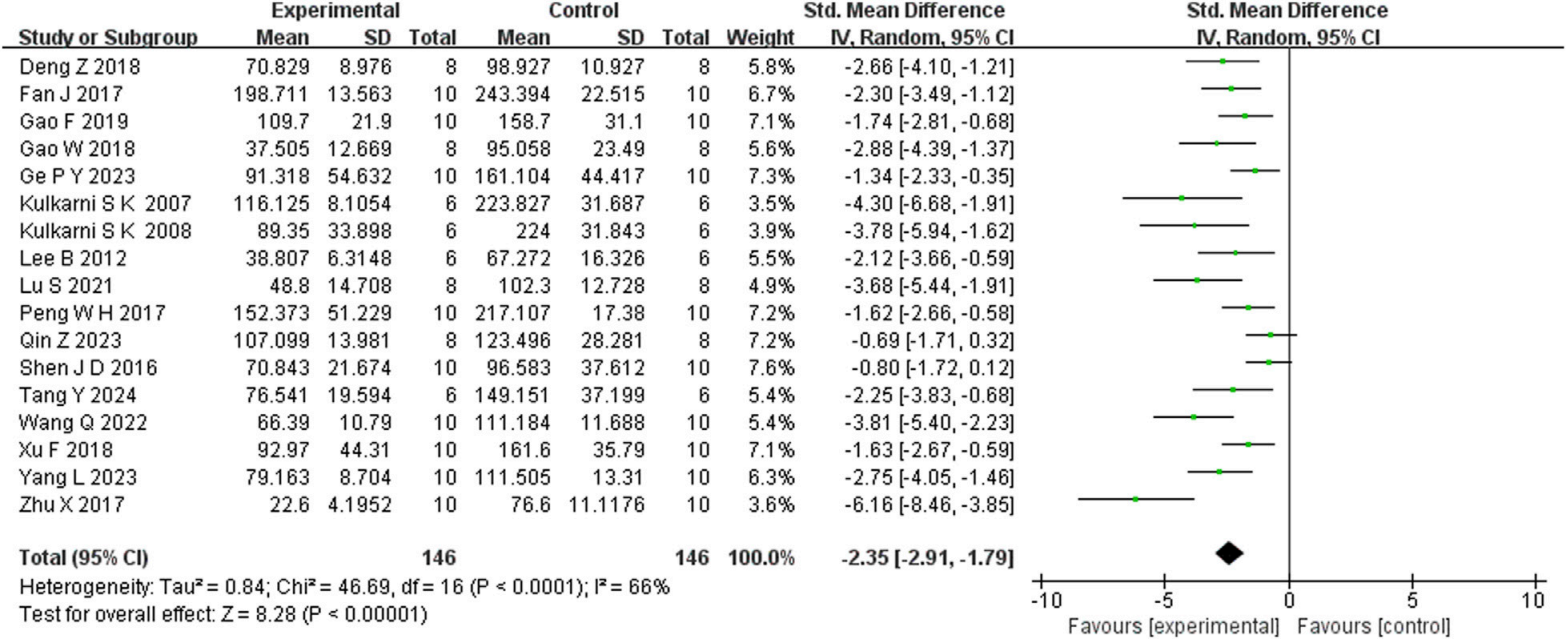
Forest plot: random effects meta-analysis of FST.

**FIGURE 6:**
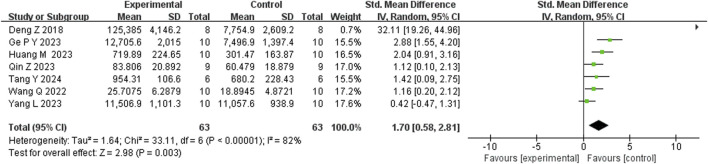
Forest plot: random effects meta-analysis of total distance in OFT.

**FIGURE 7:**
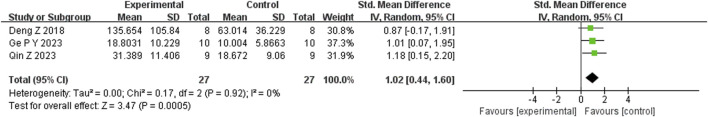
Forest plot: random effects meta-analysis of time duration in OFT.

**FIGURE 8:**
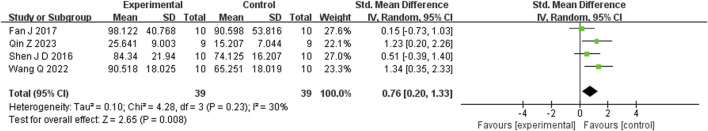
Forest plot: random effects meta-analysis of the number of crossings in OFT.

Seventeen studies reported that BBR reduced immobility time in the FST (n = 17; SMD = −2.35; 95% CI: 2.91 to −1.79; I^2^ = 82%, *P* < 0.00001; Figure 5). Seven studies showed that BBR increased total distance moved in the OFT (n = 7; SMD = 1.70; 95% CI: 0.58 to 2.81; I^2^ = 83%, *P* < 0.00001; Figure 6). Three studies indicated that BBR increased time spent in the center square of the OFT (n = 3; SMD = 1.02; 95% CI: 0.44 to 1.60; I^2^ = 0%, *P* = 0.92; Figure 7). Finally, four studies demonstrated an increase in the number of crossings in the OFT with BBR treatment (n = 4; SMD = 0.76; 95% CI: 0.20 to 1.33; I^2^ = 30%, *P* = 0.23; Figure 8).

#### 3.4.2 Inflammation indicators

This analysis included three inflammatory markers—TNF-α, IL-1β, and IL-6—reported in the 20 included studies.

**FIGURE 9:**
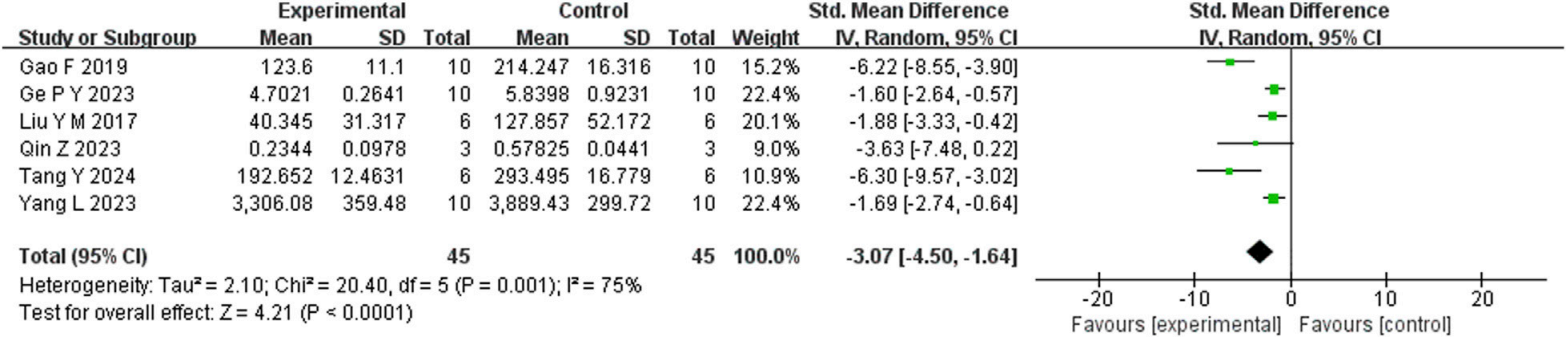
Forest plot: random effects meta-analysis of TNF-α.

**FIGURE 10:**
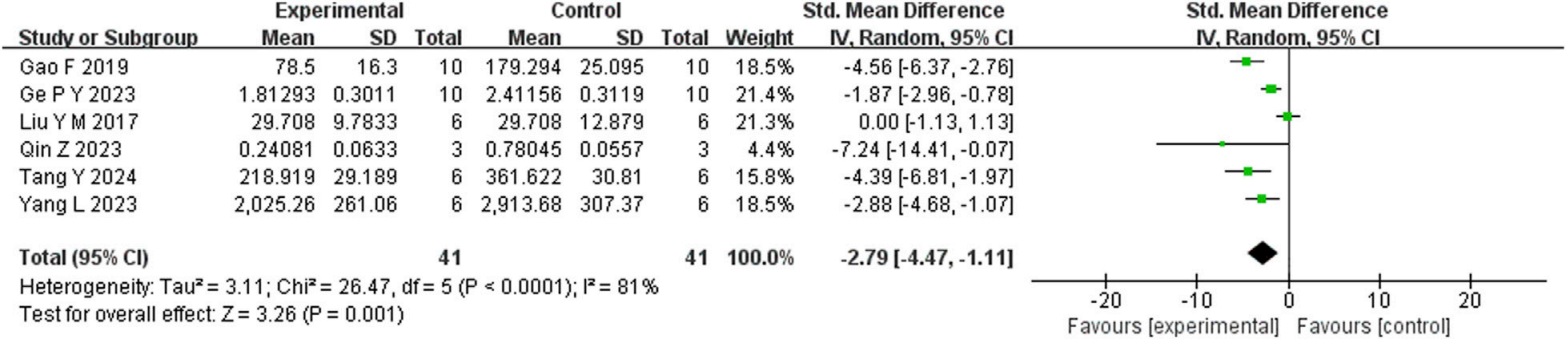
Forest plot: random effects meta-analysis of IL-1β.

**FIGURE 11:**
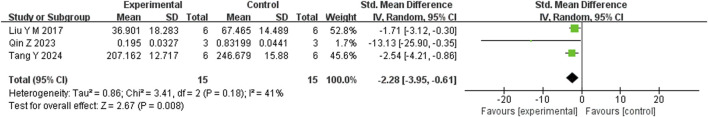
Forest plot: random effects meta-analysis of IL-6.

Six studies reported that BBR significantly reduced TNF-α levels compared to the control group (n = 6; SMD = −3.07; 95% CI: 4.50 to −1.64; heterogeneity: I^2^ = 75%, *P* = 0.001; Figure 9). Another six studies demonstrated that BBR significantly decreased IL-1β levels (n = 6; SMD = −2.79; 95% CI: 2.79 to −1.11; I^2^ = 81%, *P* < 0.00001; Figure 10). Additionally, three studies showed that BBR reduced IL-6 levels compared to controls (n = 3; SMD = −2.28; 95% CI: 3.95 to −0.61; I^2^ = 41%, *P* = 0.18; Figure 11).

#### 3.4.3 Neurotransmitters

This analysis included three neurotransmitter indicators—5-hydroxytryptamine (5-HT), norepinephrine (NE), and dopamine (DA)—reported across the 20 included studies.

**FIGURE 12:**
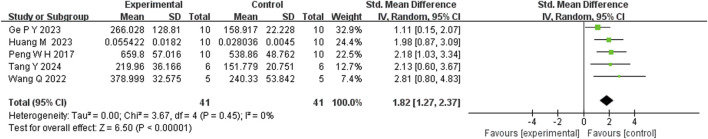
Forest plot: random effects meta-analysis of 5-HT.

**FIGURE 13:**
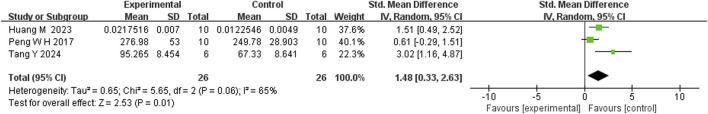
Forest plot: random effects meta-analysis of NE.

**FIGURE 14:**
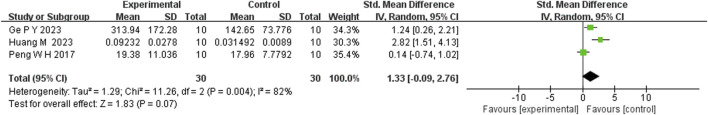
Forest plot: random effects meta-analysis of DA.

Five studies reported that berberine (BBR) increased 5-HT levels compared to the control group (n = 5; SMD = 1.82; 95% CI: 1.27 to 2.37; heterogeneity: I^2^ = 0%, *P* = 0.45; Figure 12). Three studies showed that BBR significantly elevated NE levels (n = 3; *SMD* = 1.48; 95% CI: 0.33 to 2.63; I^2^ = 65%, *P* = 0.06; Figure 13). Regarding DA levels, three studies reported minimal differences between the BBR-treated and control groups (n = 3; SMD = 1.33; 95% CI: 0.09 to 2.76; I^2^ = 82%, *P* = 0.004; Figure 14).

#### 3.4.4 Brain-derived neurotrophic factor

This analysis included two indicators—BDNF protein levels and BDNF mRNA levels—to represent changes in neuroplasticity-related outcomes reported across the 20 included studies.

**FIGURE 15:**
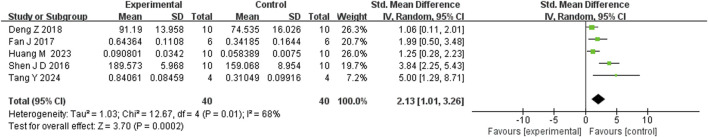
Forest plot: random effects meta-analysis of BDNA protein.

**FIGURE 16:**
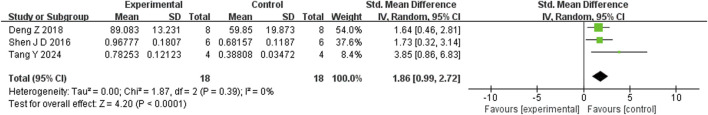
Forest plot: random effects meta-analysis of BDNA mRNA.

Four studies reported that berberine (BBR) significantly increased BDNF protein levels compared to controls (n = 4; SMD = 2.13; 95% CI: 1.01 to 3.26; heterogeneity: I^2^ = 68%, *P* = 0.01; Figure 15). An increase in BDNF mRNA levels following BBR treatment was observed in three studies (n = 3; SMD = 1.86; 95% CI: 0.99 to 2.72; I^2^ = 0%, *P* = 0.39; Figure 16).

### 3.5 Subgroup analysis

The subgroup analyses revealed that animal body weight, BBR dosage, and administration route significantly influenced specific outcomes. In the body weight subgroup, animals weighing <100 g showed larger effect sizes in the tail suspension test (SMD = −3.03 vs. −1.36, P-between = 0.025) and BDNF protein levels, while the ≥100 g subgroup demonstrated greater effects in dopamine levels (P-between = 0.013). In the dosage subgroup, lower BBR dosage (<100 mg/kg) produced greater effects on body weight (SMD = 4.64, P-between <0.001), while higher dosage (>100 mg/kg) showed stronger effects on TNF-α reduction (SMD = −5.16, P-between = 0.025). Significant dopamine improvement was only observed at 100 mg/kg (SMD = 2.82). Regarding administration routes, intragastric administration was most effective for IL-1β reduction (P-between <0.001) while gavage administration showed the greatest effects on dopamine levels (P-between = 0.044). Most behavioral tests showed no significant subgroup differences. Considerable heterogeneity (I^2^ > 50%) was observed in several subgroups (detailed results provided in Supplementary Figures S17–S55).

### 3.6 Publication bias

Publication bias was assessed using two approaches. For outcomes reported in more than 10 studies, both Begg’s test and Egger’s test were performed. Additionally, sensitivity analyses were conducted for these outcomes. For indicators reported in fewer than 10 studies, no further bias analysis was conducted.

#### 3.6.1 Begg’s test and Egger’s test

Two indicators—sucrose preference test (SPT) and immobility time in the forced swim test (FST)—were reported in more than 10 studies. Therefore, Begg’s test and Egger’s test were conducted to assess potential publication bias. For the SPT, Begg’s test indicated significant publication bias (*P* < 0.0005; *P* = 0.004). Similarly, both Begg’s test and Egger’s test for immobility time in the FST also indicated significant publication bias (Begg’s test: *P* < 0.0005; *P* < 0.001; Egger’s test: *P* < 0.0005; *P* = 0.000). These results suggest the presence of significant publication bias in these indicators.

#### 3.6.2 Sensitivity analysis

Meanwhile, sensitivity analysis was conducted for 2 indicators (SPT, Immobility time in FST) (the details in Supplementary Figures S56, S57). The analysis indicates that the 2 experimental results exhibit a certain degree of robustness.

## 4 Discussion

The aim of this study was to synthesize preclinical studies to evaluate the efficacy and potential mechanisms of BBR in the treatment of depression. Our findings provide further evidence of BBR’s effectiveness in promoting weight gain and producing antidepressant-like effects in animal models. We also found that BBR reduces levels of inflammatory markers (IL-6, IL-1β, TNF-α), enhances neurotransmitter levels (5-HT, NE—but not DA), and increases neuroprotective factors, including BDNF protein and BDNF mRNA expression.

The results suggest that BBR may increase body weight and modulate various depression-like behaviors, indicating its potential to alleviate different depressive symptoms to varying degrees.

Though, patients with depression exhibit heterogeneity in body weight changes. In this study, depressive model animals exhibited the expected reduction in body weight, while BBR treatment restored their body weight to within the normal control range. This “restorative increase” in body weight, which’s it to normal levels, suggests that BBR may exert its therapeutic effects by correcting depression-related physiological disturbances, such as appetite loss. Furthermore, not all studies in our review reported weight gain following BBR administration (Shen et al., 2016), possibly due to the non-dose-dependent nature of BBR’s antidepressant effects (Kulkarni and Dhir, 2007; Kulkarni and Dhir, 2008) and our subgroup analysis based on animal body weight further corroborates this observation. Reduced sucrose preference is widely used as a proxy for anhedonia, a core symptom of depression (Riaz et al., 2015; Willner, 2005). The observed enhancement of sucrose preference following BBR treatment suggests a potential benefit for anhedonia. In previous research, the FST and tail TST have been validated as predictors of antidepressant activity (Kulkarni and Dhir, 2007) and are associated with behavioral despair (Xing et al., 2019). In our analysis, BBR significantly reduced immobility time in both tests, indicating its ability to mitigate despair-like behaviors. In contrast to previous reports (Kulkarni and Dhir, 2007; Kulkarni and Dhir, 2008), this effect demonstrated no association with a linear dose-response relationship but was significantly influenced by animal body weight.

Furthermore, several OFT indicators—including total distance traveled, time spent in the center, and number of crossings—are commonly interpreted as behavioral responses to psychotropic treatments (Schulz et al., 2023). Our findings suggest that BBR administration improves anxiety- and fear-related behaviors (Kraeuter et al., 2019; Walz et al., 2016). Collectively, these results support the potential of BBR for future clinical use in the prevention and treatment of depressive disorders.

In terms of mechanisms, BBR has been shown to influence three major biological systems relevant to depression. However, the underlying mechanisms remain complex and not fully understood. One of the leading hypotheses for the pathophysiology of depression involves inflammatory pathways, first proposed in 1987 (Renault et al., 1987). Increasing evidence suggests that both peripheral and central inflammation contribute significantly to the risk and susceptibility to depression (Beurel et al., 2020; Colasanto et al., 2020; Enache et al., 2019). Pro-inflammatory cytokines such as IL-1β, IL-6, and TNF-α are widely recognized as classic biomarkers of inflammation (Felger and Lotrich, 2013). Moreover, the relationship between inflammation and depression appears to be biphasic (Beurel et al., 2020; Kiecolt-Glaser et al., 2015). Notably, some antidepressants have also demonstrated anti-inflammatory effects (Johnston et al., 2023; Köhler et al., 2018). Although a few studies have suggested that BBR may exacerbate the inflammatory response (Zhu and Qian, 2006), our meta-analysis aligns with major previous reports (Gong et al., 2024; Jeong et al., 2009) by supporting the anti-inflammatory role of BBR in regulating typical pro-inflammatory cytokines such as IL-6, IL-1β, and TNF-α.

Despite these findings, the precise mechanisms by which BBR exerts its anti-inflammatory effects remain unclear. Recent studies have implicated several molecular pathways and regulators, including acetylation of p65 at Lys310 by p300 in macrophages (Zhang et al., 2023), EIF2AK2 (Wei et al., 2023), the NF-κB signaling pathway (Yu et al., 2019; Tang et al., 2021), the NLRP3 inflammasome pathway (Yang et al., 2023), and the ADK/AMPK/Nrf2 signaling axis (Cheng et al., 2024). Additionally, BBR may help maintain immunodynamic homeostasis through multiple immune-related mechanisms (Vita and Pullen, 2022; Wang et al., 2020; Xia et al., 2024).

Secondly, it is widely believed that depression results from an imbalance of 5-HT, NE, DA, or other neurochemical substances in the brain (Pilkington et al., 2018). However, our findings partially contradict previous studies (Mohi-Ud-Din et al., 2022), as DA levels did not increase following BBR treatment. Our subgroup analysis confirms the non-dose-dependent pharmacology of BBR reported in previous studies (Huang et al., 2023; Peng et al., 2007): DA levels increased significantly only at the 100 mg/kg dosage. This finding underscores the importance of methodological considerations in interpreting BBR’s complex interactions with neurotransmitter systems. Different doses of BBR may exert varying effects on specific neurotransmitters (Arora and Chopra, 2013; Kulkarni and Dhir, 2008). For instance, BBR has been shown to inhibit monoamine oxidase (Peng et al., 2007) and influence organic cation transporter 2 and 3 activity (Sun et al., 2014), which may contribute to increased levels of certain neurotransmitters. The BBR-mediated enhancement of neurotransmitters may represent one step in a broader cascade of events leading to its antidepressant effects (Arora and Chopra, 2013). Moreover, recent studies suggest that BBR may exert synergistic effects when combined with classical antidepressants (Sun et al., 2014).

Notably, BBR is capable of rapidly crossing the blood–brain barrier to exert neuroprotective effects (Kulkarni and Dhir, 2010; Tian et al., 2023; L. Wang et al., 2021; Yang et al., 2018). The relationship between BBR and BDNF has been increasingly studied over the past decade. BDNF is a key neurotrophin widely distributed in the brain and is essential for neuronal survival and plasticity (Zhang et al., 2016). It has been demonstrated that one of the mechanisms through which BBR exerts its effects is by increasing BDNF levels, thereby promoting neuronal nourishment, conferring anti-seizure activity (Jivad et al., 2024), preventing neurodegeneration (Begh et al., 2025), and offering cognitive protection (Begh et al., 2025; Shaker et al., 2021). Furthermore, BBR’s effect on BDNF expression resembles that of certain antidepressants (Hess et al., 2022). This regulatory effect may occur through modulation of the PI3K/AKT signaling pathway (Tang et al., 2024), inhibition of the NF-κB signaling pathway (Yu et al., 2019), and activation of the cAMP response element-binding protein (Tang et al., 2024; Yu et al., 2019). These pathways ultimately influence BDNF expression. However, the precise molecular mechanisms by which BBR upregulates BDNF remain unclear. Importantly, BDNF has also been implicated in the regulation of inflammatory responses (Kim et al., 2023), further supporting BBR’s potential anti-inflammatory effects. Additionally, some studies have shown that BDNF may enhance the levels of neurotransmitters (Bastioli et al., 2022), highlighting its broader role in neuropsychiatric regulation.

### 4.1 Advantages

There is an urgent need for new antidepressant therapies. This study provides a comprehensive summary of preclinical findings on the effects of BBR in the treatment of depression, offering foundational evidence to support its potential therapeutic use. Although the exact mechanisms underlying BBR’s antidepressant-like effects remain unclear and were not definitively established in this study, our findings represent an important step forward. This work offers both theoretical insights and practical guidance for future research on BBR, potentially accelerating the development of novel antidepressant agents targeting diverse etiologies of depression (Xu et al., 2018).

### 4.2 Limitation

Several important considerations should be noted before interpreting the findings of this study:

1. The near-exclusive use of male animals in the included studies limits the generalizability of our findings to both sexes, as it fails to account for potential sex-based differences. This constraint necessitates caution when extrapolating the results to clinical settings, particularly given the well-documented sex-specific characteristics of depression (Salk et al., 2017).
2. Prior studies have shown that inflammatory markers, neurotransmitters, and BDNF interact with one another (Bastioli et al., 2022; Hodo et al., 2020; Kim et al., 2023; Oshaghi et al., 2023; Zhu et al., 2022). In our study, it remains unclear whether the observed effects are due to these interactions or if causal relationships exist among these factors. Further research is needed to clarify these complex linkages.
3. While this study contributes a novel perspective on the treatment of depression, the mechanism by which BBR exerts its antidepressant effects is still not fully understood, and current findings are limited to animal models. Before BBR can be translated into clinical practice, it is crucial to recognize that animal experimental results cannot be directly extrapolated to humans. Three key issues require resolution: species differences preventing direct translation of effective dosage, unknown drug interaction mechanisms, and unverified long-term safety profiles. These inherent limitations determine that the current findings can only serve as reference for subsequent clinical work.
4. This meta-analysis is limited by substantial heterogeneity, reflecting methodological variations in BBR sources, animal models, administration routes, and detection methods among included studies. The findings should therefore be interpreted as representing a range of potential effects under different experimental conditions. Future studies would benefit from standardized protocols and complete methodological reporting to improve evidence synthesis.
5. This study has important limitations, including concerns regarding the high risk of bias in most included studies and the presence of publication bias, as evidenced by significant Begg’s and Egger’s tests for SPT and FST (P < 0.05). These issues call for cautious interpretation of the findings.

## 5 Conclusion

In summary, our analysis indicates that BBR may be effective in reducing depression-like behaviors across various animal models. Moreover, the findings suggest that BBR has the potential to modulate inflammatory factors, neurotransmitters, and BDNF. However, further investigation is needed to elucidate the complex mechanisms through which BBR regulates these systems and to determine whether additional interrelationships exist among them. Importantly, it remains unclear whether the effects observed in preclinical studies can be replicated in clinical settings, and the safety profile of BBR in humans warrants further exploration. Therefore, more rigorous and comprehensive evidence is required to support the translation of these findings into clinical practice and to realize potential therapeutic benefits for patients with depression.

## Data Availability

The original contributions presented in the study are included in the article/Supplementary Material, further inquiries can be directed to the corresponding author.

## Abbreviations

5-HT, 5-hydroxytryptamine; BBR, berberine; BDNF, brain-derived neurotrophic factor; CI, confidence interval; CIs, confidence intervals; DA, dopamine; FST, forced swim test; IL-1β, Interleukin-1β; IL-6, Interleukin 6; NE, Norepinephrine; OFT, open field test; SD, standard deviation; SMD, standardized mean difference; SMDs, standardized mean differences; SPT, Sucrose preference in sucrose preference test; TNF-α, Tumor necrosis factor α; TST, Tail suspension test.
