# Supplementary material for: The potential value of the use of berberine in depression: a systematic review and meta-analysis of preclinical studies
Source: Front Pharmacol. 2025 Nov 3;16:1664784. doi: 10.3389/fphar.2025.1664784 (PMC12620832; doi:10.3389/fphar.2025.1664784)
Supplement: Supplementary file 1 [file DataSheet1.zip › Supplementary Figures/figure title.docx]

Fig19. Subgroup analysis of weight by animal body weight

Fig20. Subgroup analysis of TST by animal body weight

Fig21. Subgroup analysis of SPT by animal body weight

Fig22. Subgroup analysis of FST by animal body weight

Fig23. Subgroup analysis of total distance in OFT by animal body weight

Fig24. Subgroup analysis of the number of crossings in OFT by animal body weight

Fig25. Subgroup analysis of TNF-α by animal body weight

Fig26. Subgroup analysis of IL-1β by animal body weight

Fig27. Subgroup analysis of IL-6 by animal body weight

Fig28. Subgroup analysis of 5-HT by animal body weight

Fig29. Subgroup analysis of NE by animal body weight

Fig30. Subgroup analysis of DA by animal body weight

Fig31. Subgroup analysis of BDNA protein by animal body weight

Fig32. Subgroup analysis of weight by BBR dosage

Fig33. Subgroup analysis of TST by BBR dosage

Fig34. Subgroup analysis of SPT by BBR dosage

Fig35. Subgroup analysis of FST by BBR dosage

Fig36. Subgroup analysis of total distance in OFT by BBR dosage

Fig37. Subgroup analysis of the number of crossings in OFT by BBR dosage

Fig38. Subgroup analysis of TNF-α by BBR dosage

Fig39. Subgroup analysis of IL-1β by BBR dosage

Fig40. Subgroup analysis of IL-6 by BBR dosage

Fig41. Subgroup analysis of 5-HT by BBR dosage

Fig42. Subgroup analysis of DA by BBR dosage

Fig43. Subgroup analysis of BDNA protein by BBR dosage

Fig44. Subgroup analysis of BDNA mRNA by BBR dosage

Fig45. Subgroup analysis of weight by route of administration

Fig46. Subgroup analysis of TST by route of administration

Fig47. Subgroup analysis of SPT by route of administration

Fig48. Subgroup analysis of FST by route of administration

Fig49. Subgroup analysis of total distance in OFT by route of administration

Fig50. Subgroup analysis of time duration in OFT by route of administration

Fig51. Subgroup analysis of the number of crossings in OFT by route of administration

Fig52. Subgroup analysis of TNF-α by route of administration

Fig53. Subgroup analysis of IL-1β by route of administration

Fig54. Subgroup analysis of 5-HT by route of administration

Fig55. Subgroup analysis of DA by route of administration

Fig56. Subgroup analysis of BDNA protein by route of administration

Fig57. Subgroup analysis of BDNA mRNA by route of administration
