# Supplementary figures and images for: The potential value of the use of berberine in depression: a systematic review and meta-analysis of preclinical studies
Source: Front Pharmacol. 2025 Nov 3;16:1664784. doi: 10.3389/fphar.2025.1664784 (PMC12620832; doi:10.3389/fphar.2025.1664784)

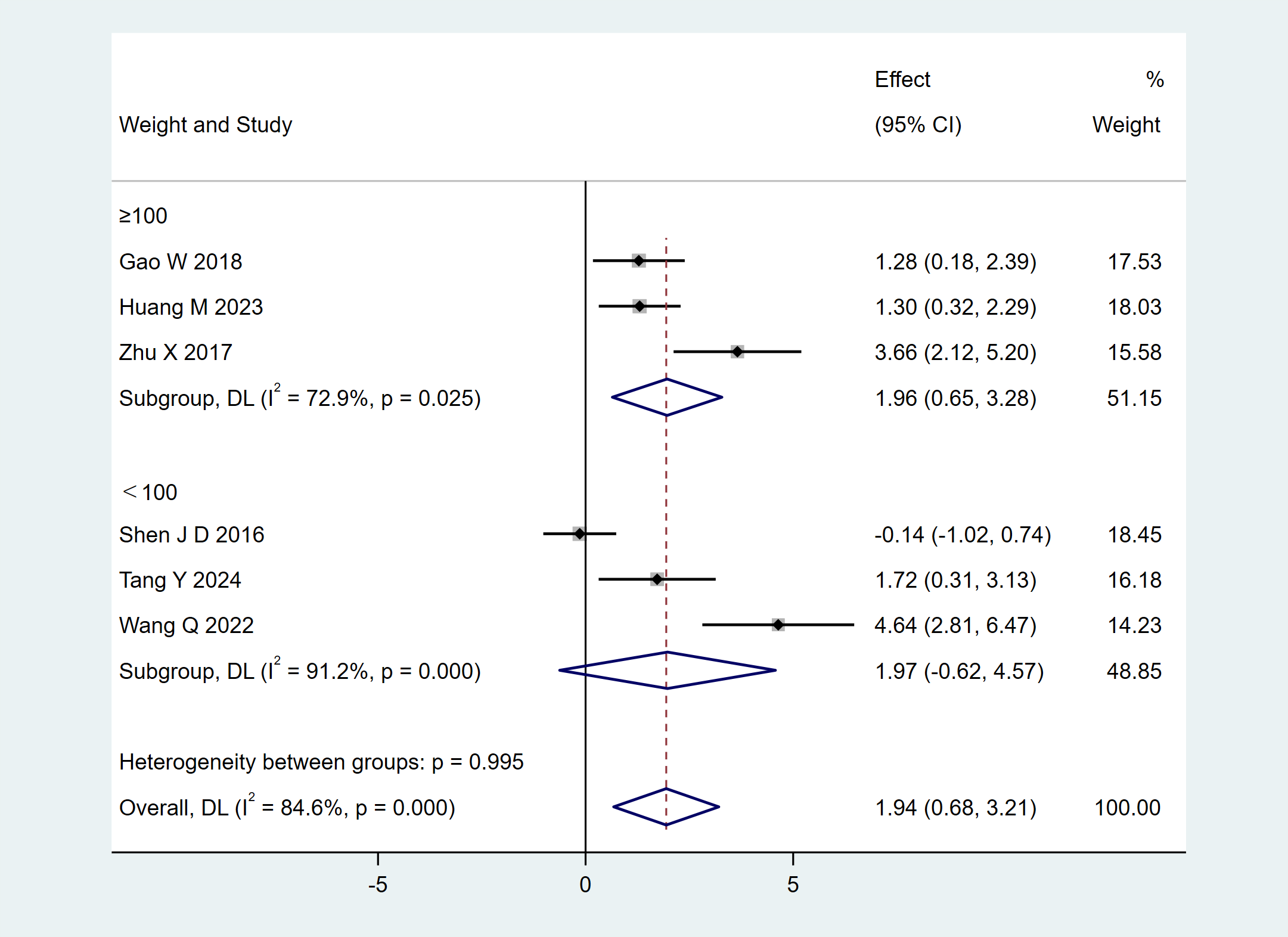

Supplement: Supplementary file 1 [file DataSheet1.zip › Supplementary Figures/Fig19.tif]

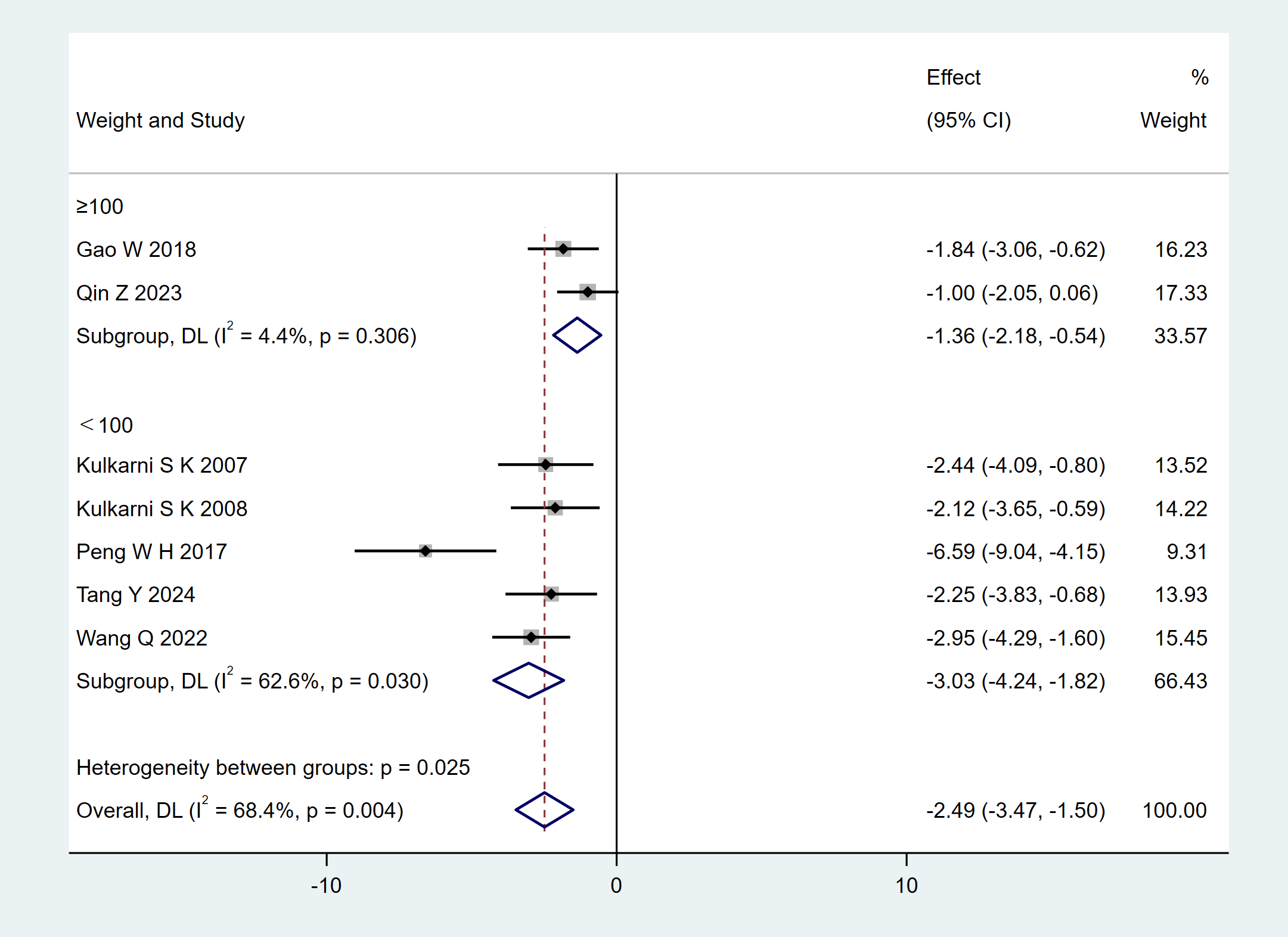

Supplement: Supplementary file 1 [file DataSheet1.zip › Supplementary Figures/Fig20.tif]

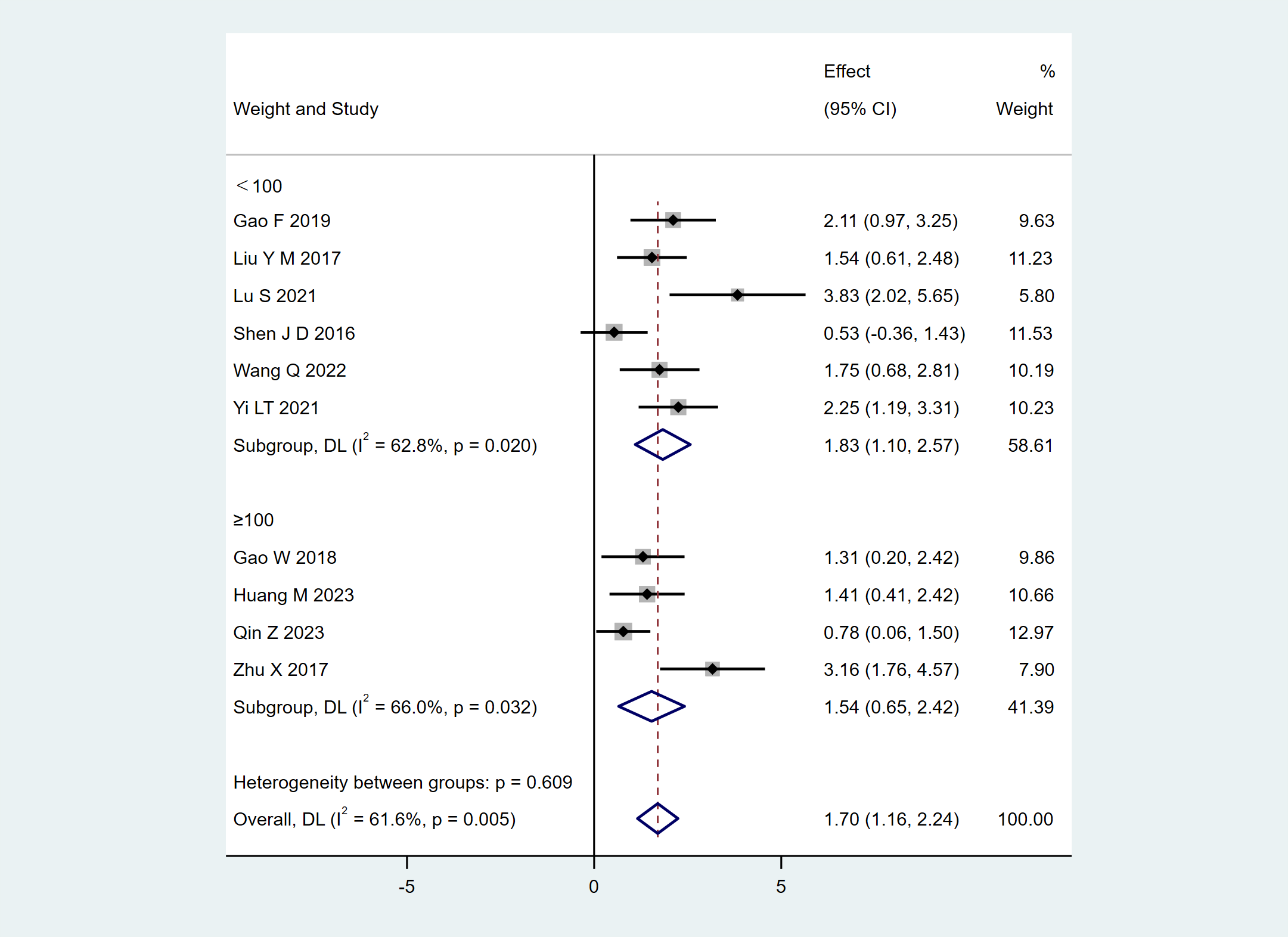

Supplement: Supplementary file 1 [file DataSheet1.zip › Supplementary Figures/Fig21.tif]

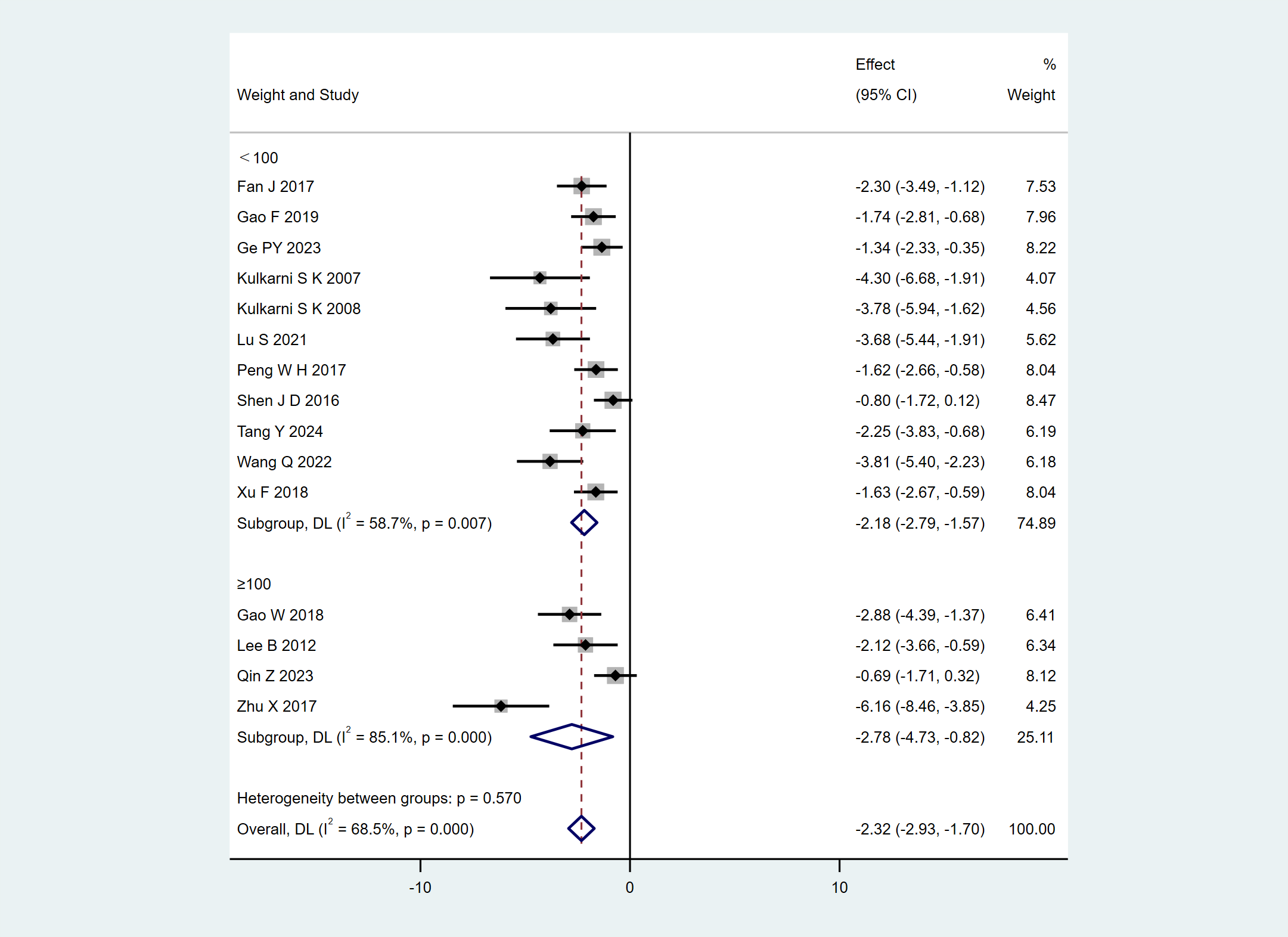

Supplement: Supplementary file 1 [file DataSheet1.zip › Supplementary Figures/Fig22.tif]

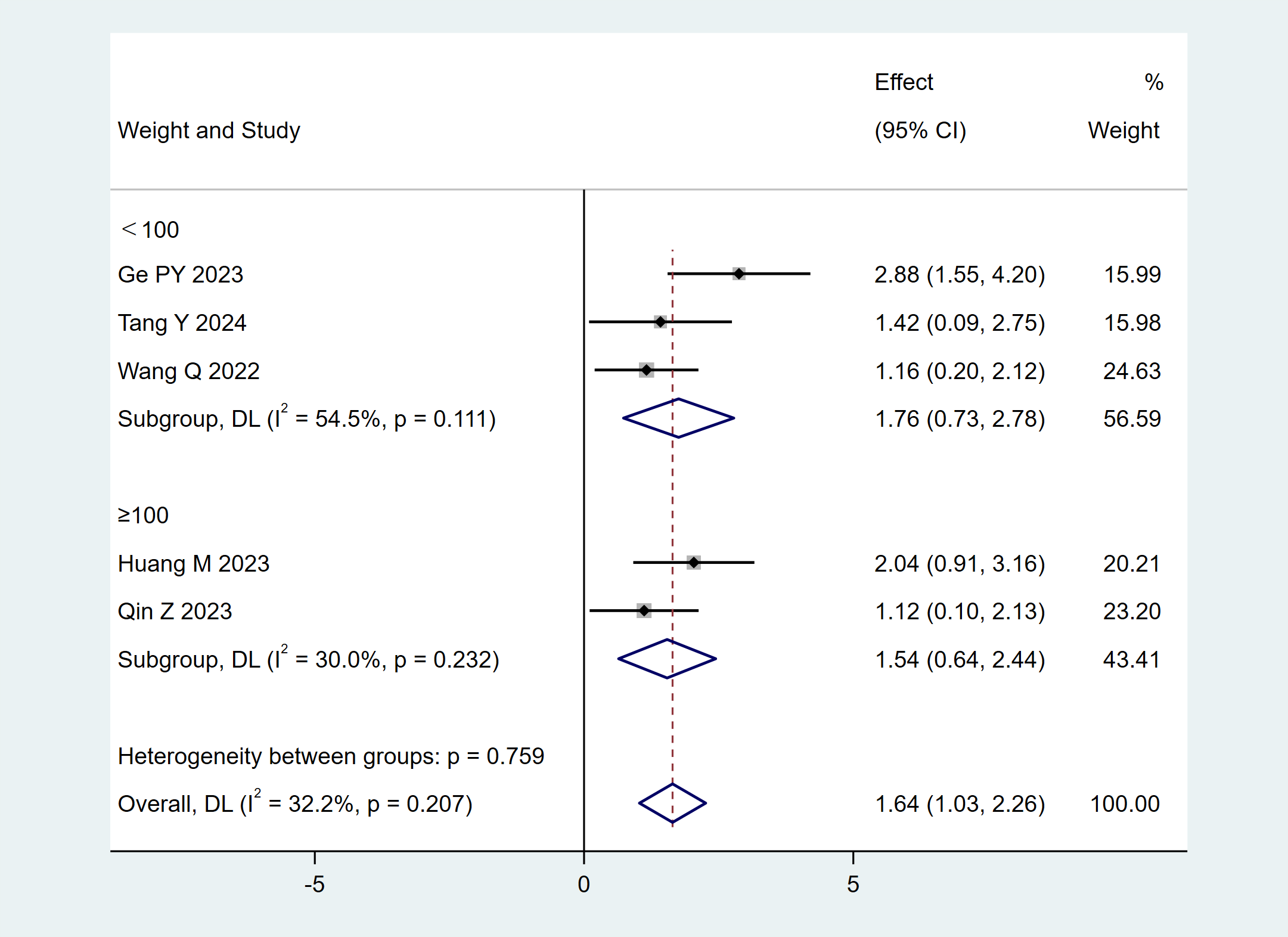

Supplement: Supplementary file 1 [file DataSheet1.zip › Supplementary Figures/Fig23.tif]

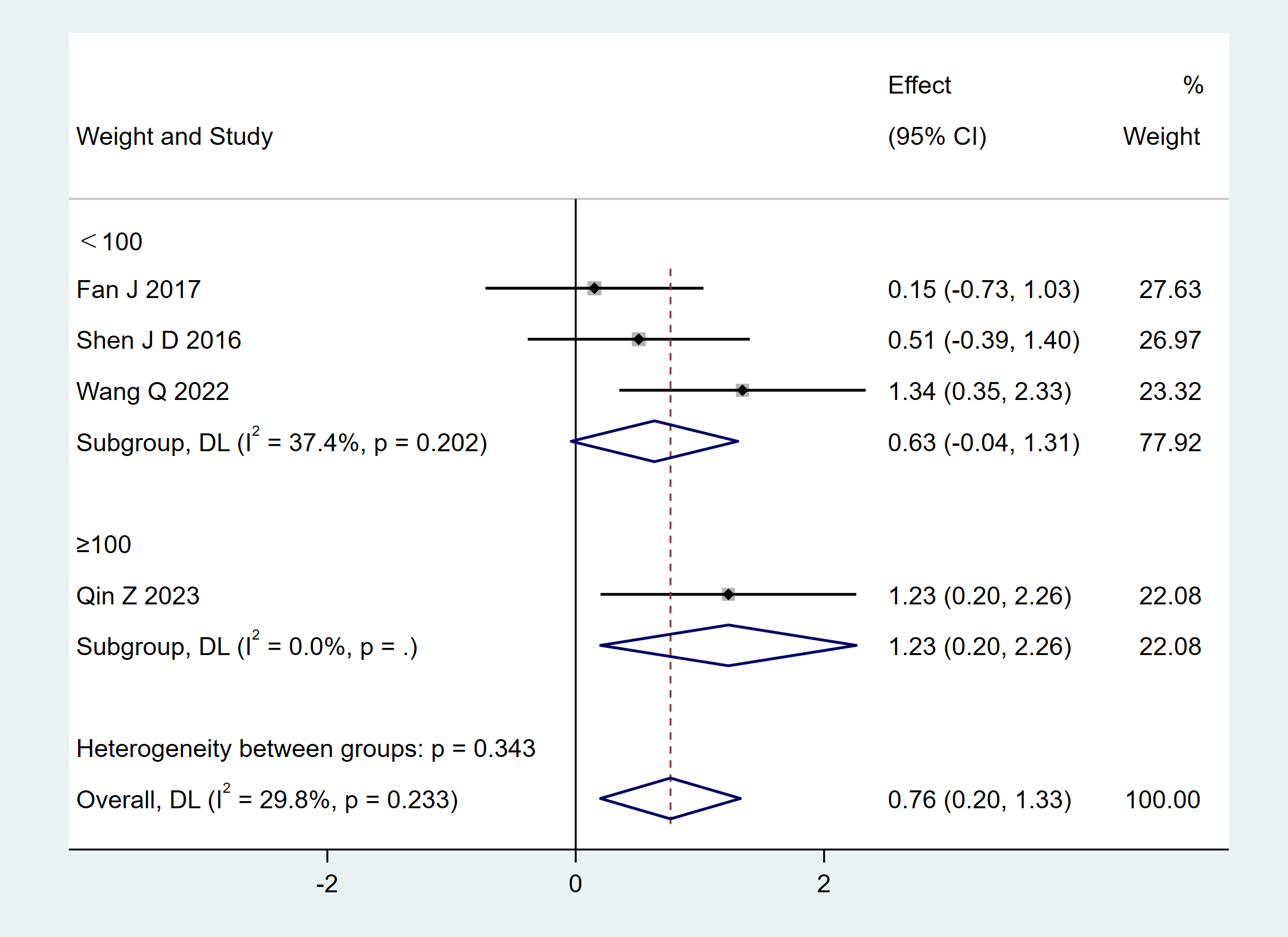

Supplement: Supplementary file 1 [file DataSheet1.zip › Supplementary Figures/Fig24.tif]

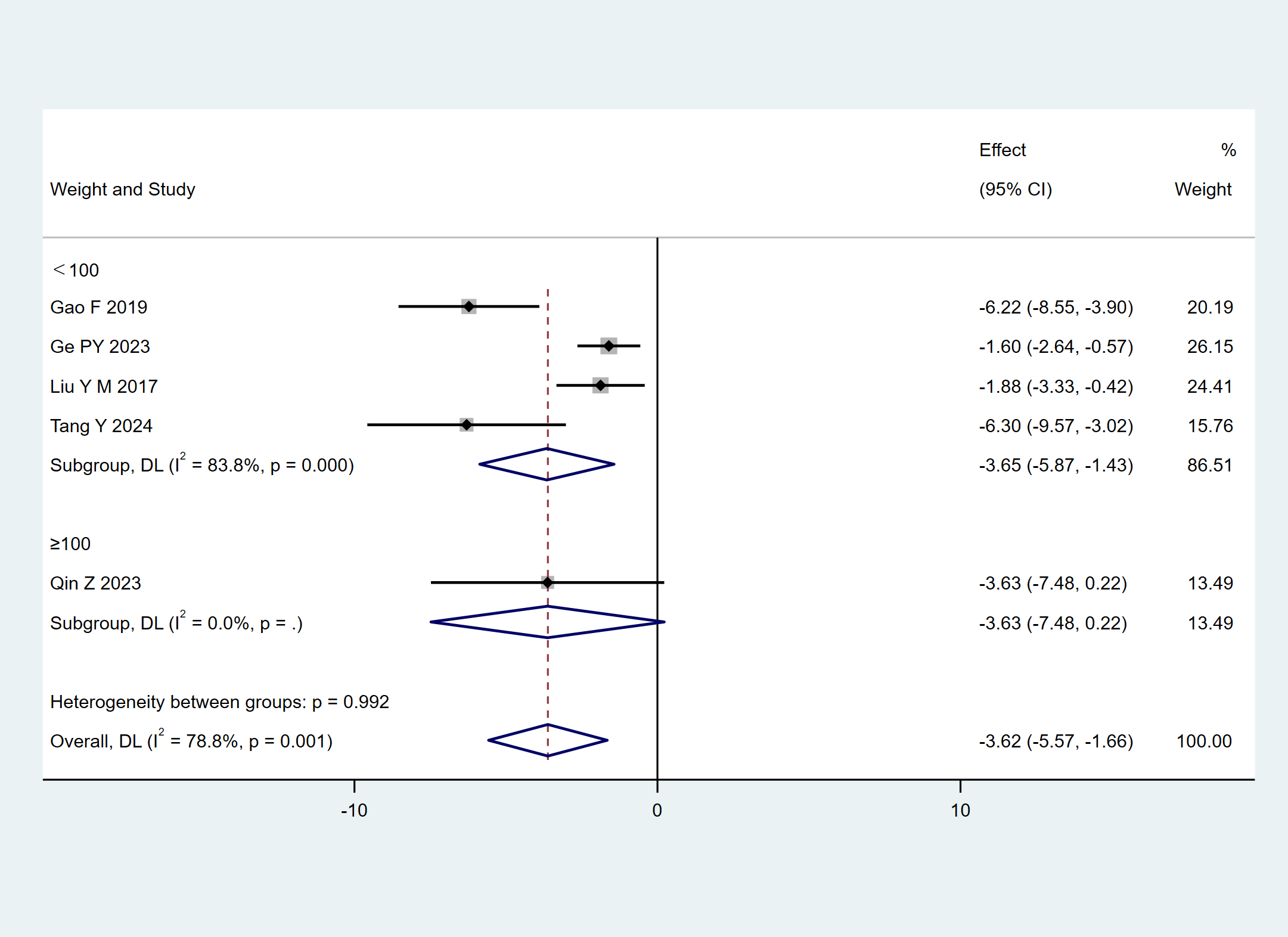

Supplement: Supplementary file 1 [file DataSheet1.zip › Supplementary Figures/Fig25.tif]

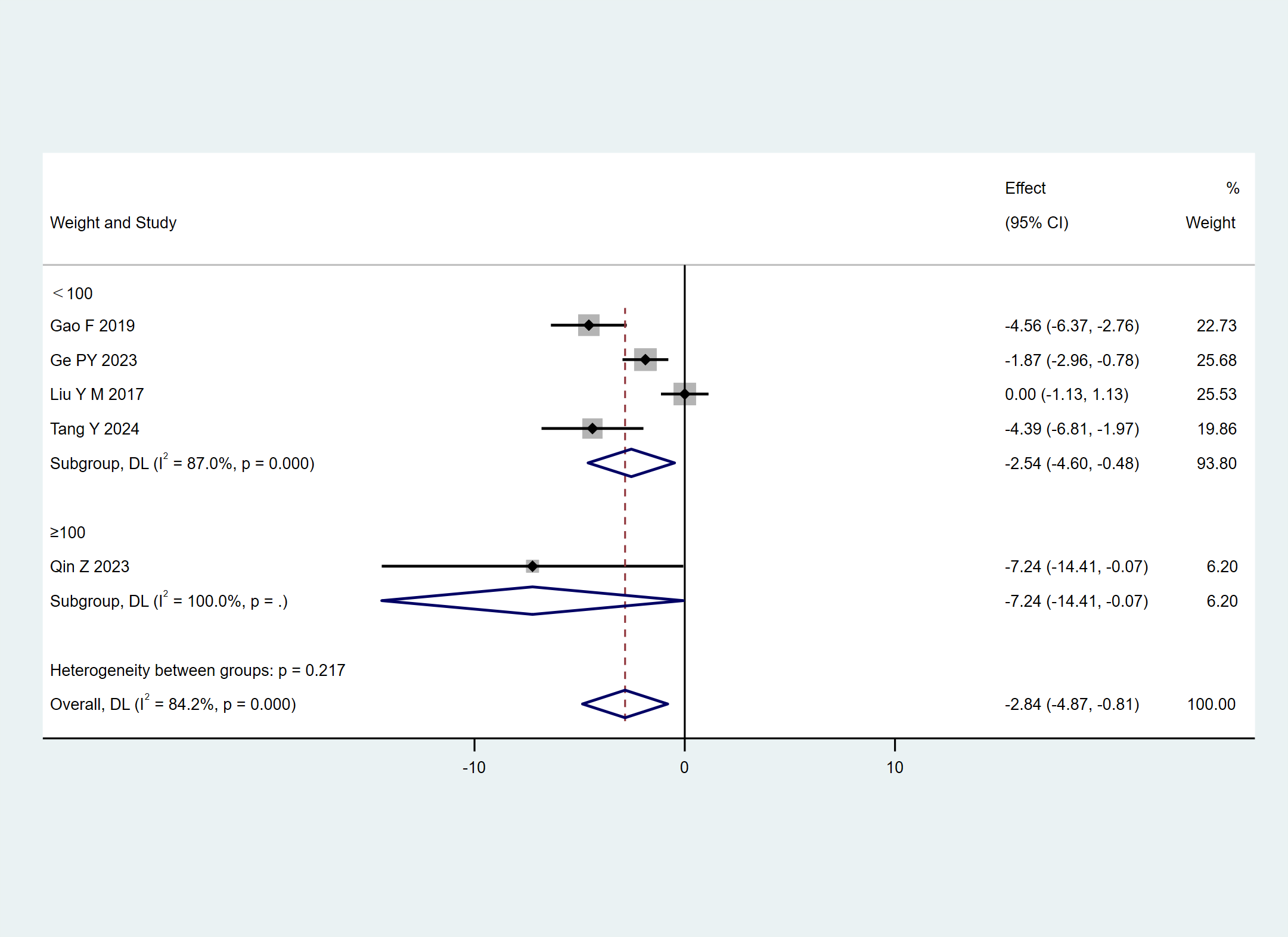

Supplement: Supplementary file 1 [file DataSheet1.zip › Supplementary Figures/Fig26.tif]

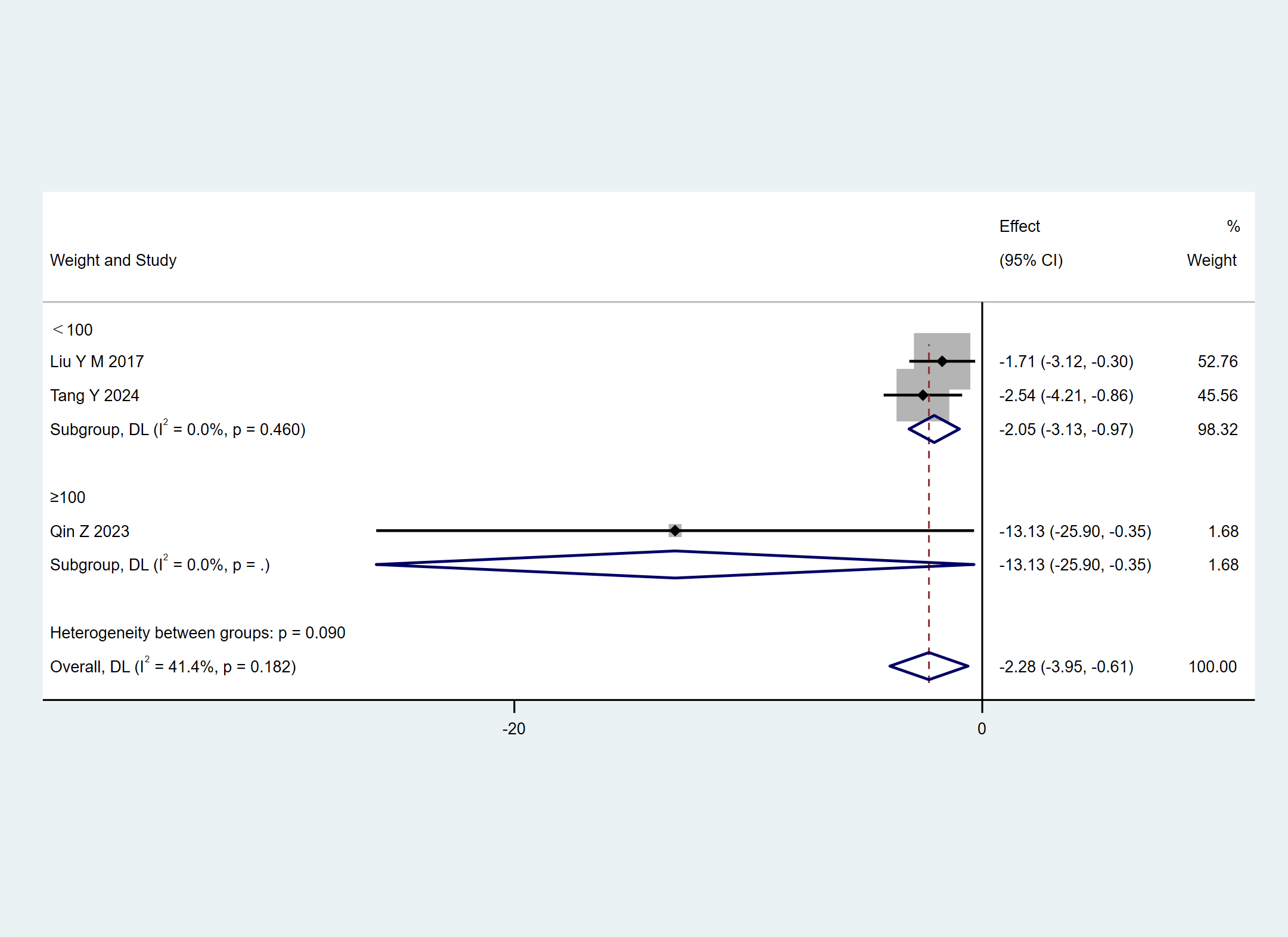

Supplement: Supplementary file 1 [file DataSheet1.zip › Supplementary Figures/Fig27.tif]

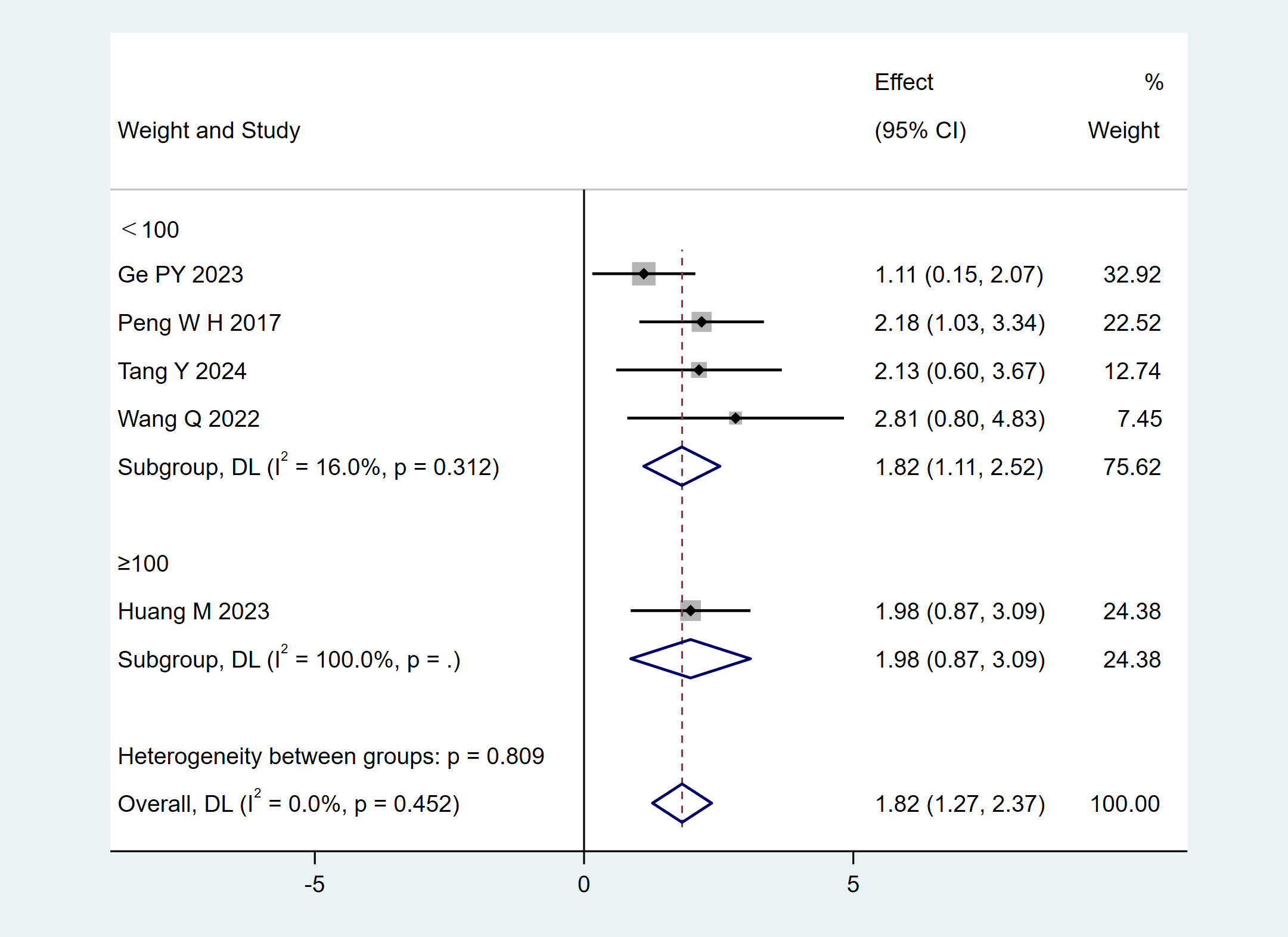

Supplement: Supplementary file 1 [file DataSheet1.zip › Supplementary Figures/Fig28.tif]

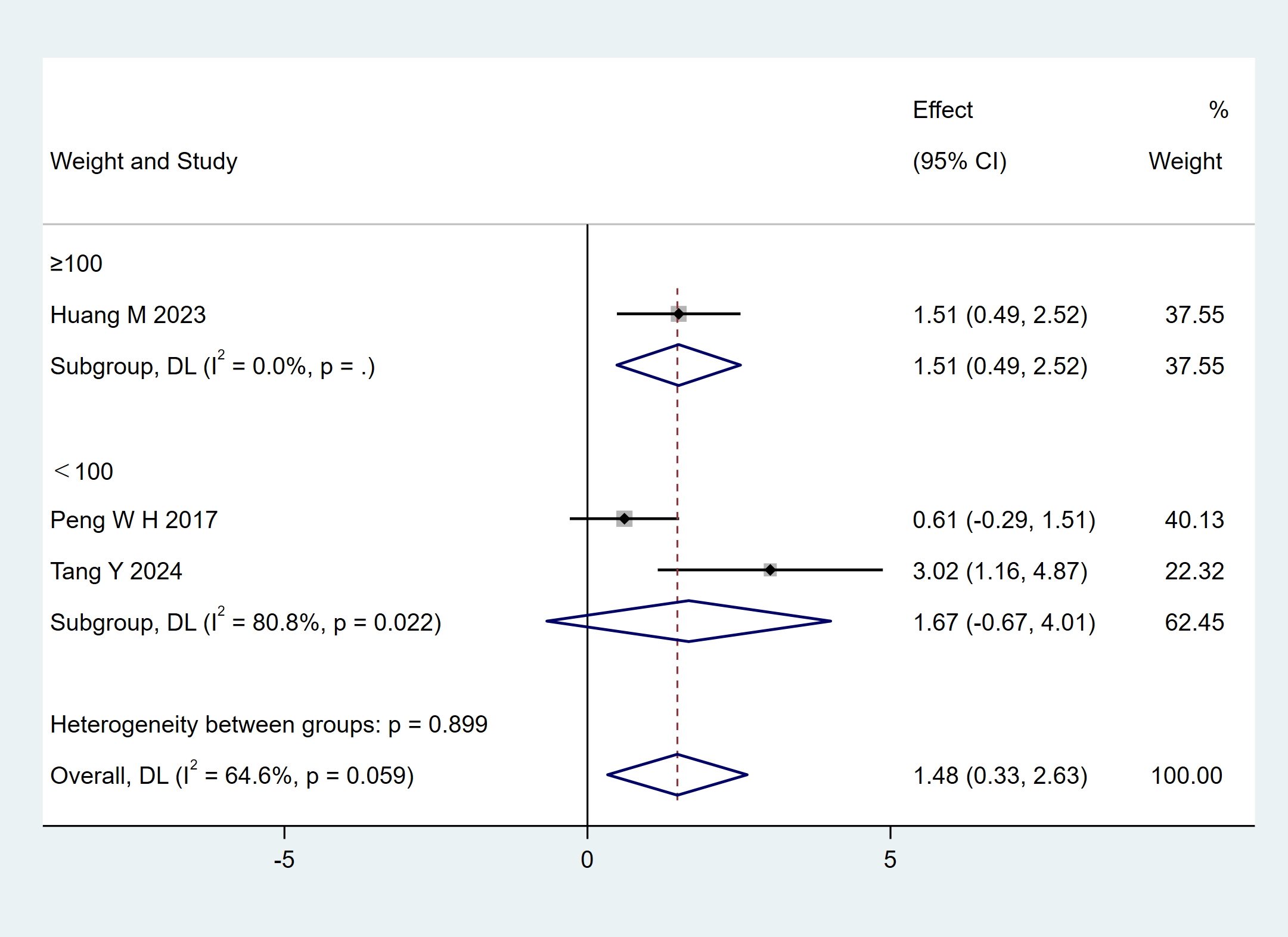

Supplement: Supplementary file 1 [file DataSheet1.zip › Supplementary Figures/Fig29.tif]

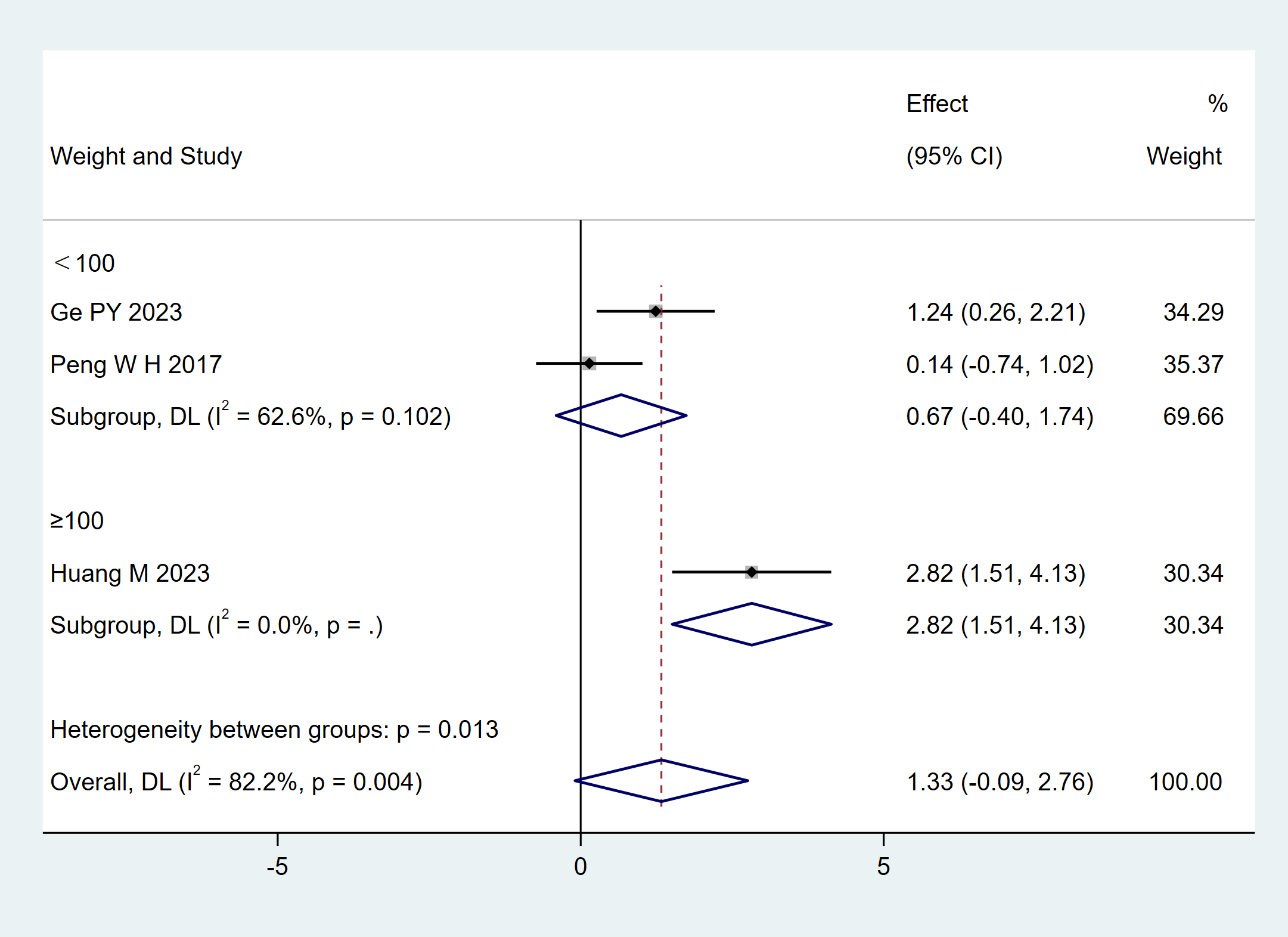

Supplement: Supplementary file 1 [file DataSheet1.zip › Supplementary Figures/Fig30.tif]

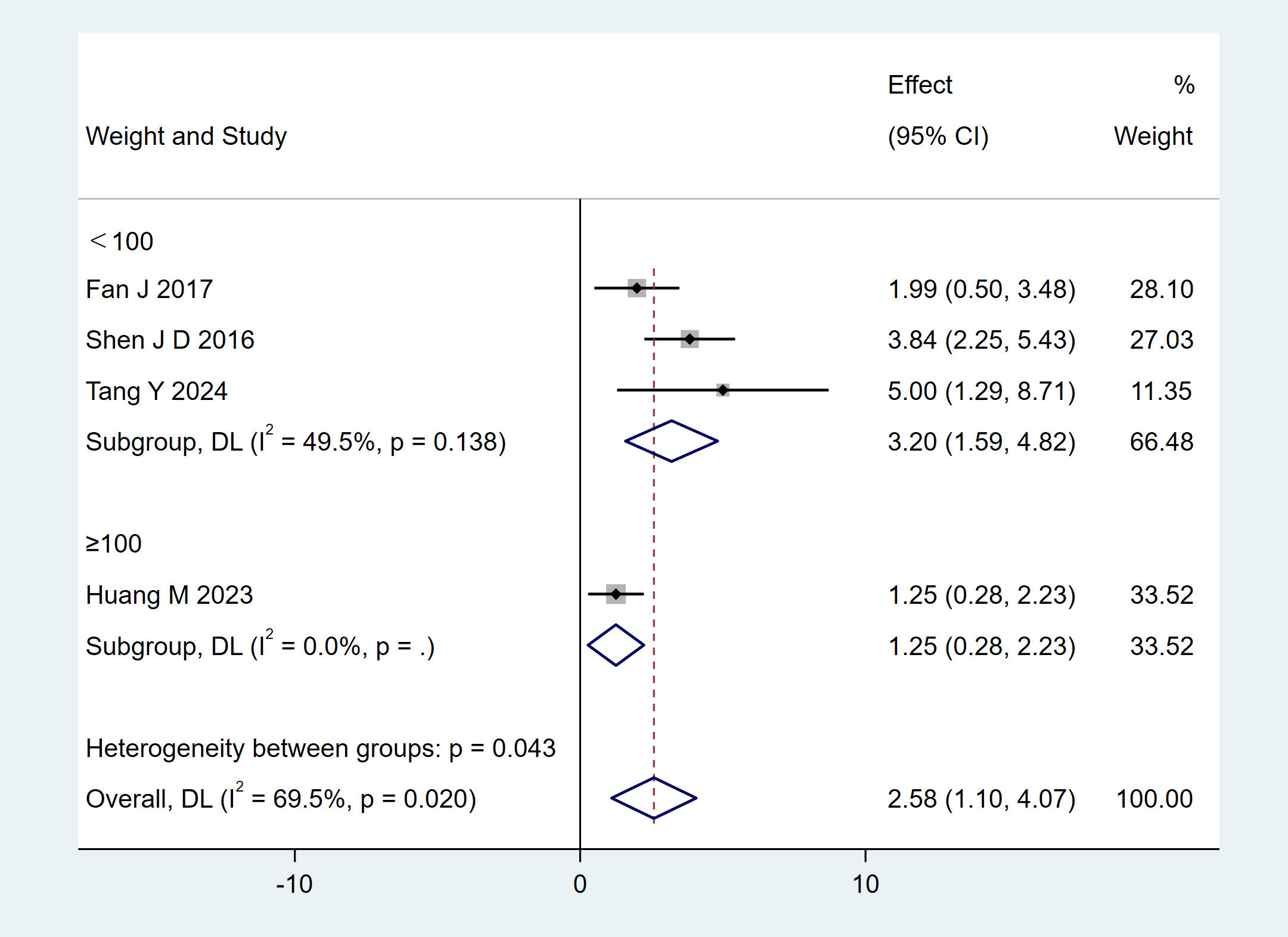

Supplement: Supplementary file 1 [file DataSheet1.zip › Supplementary Figures/Fig31.tif]

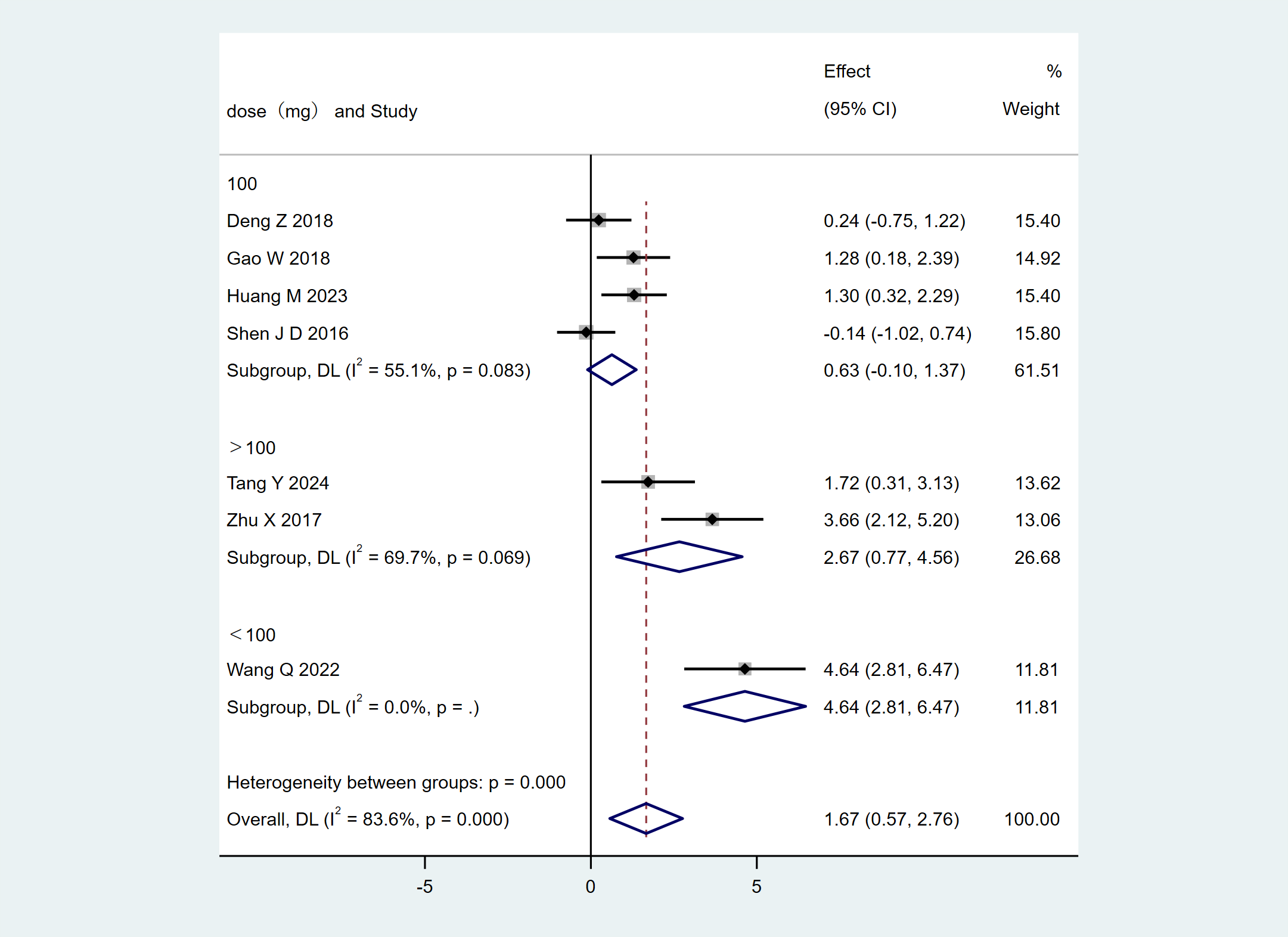

Supplement: Supplementary file 1 [file DataSheet1.zip › Supplementary Figures/Fig32.tif]

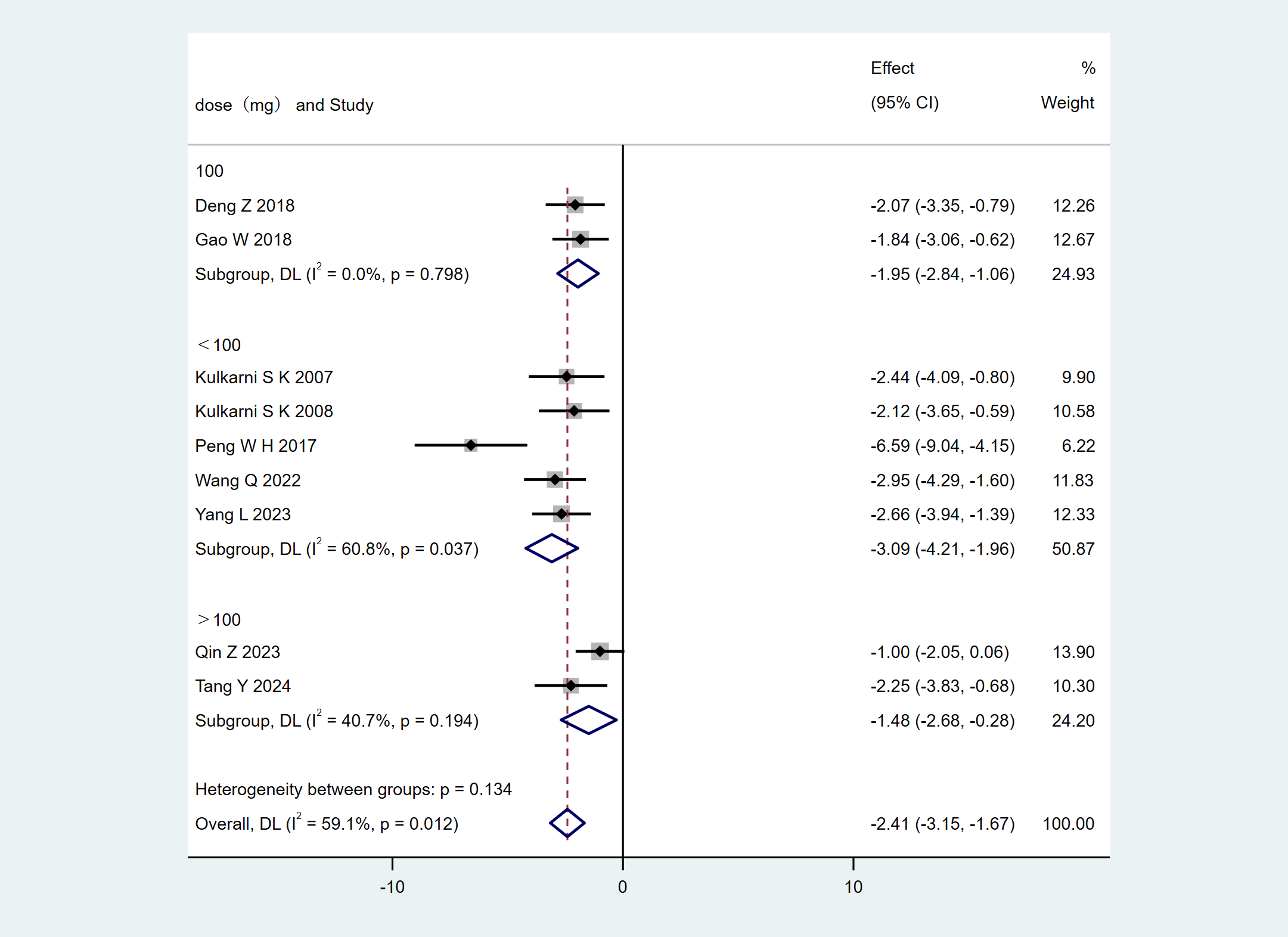

Supplement: Supplementary file 1 [file DataSheet1.zip › Supplementary Figures/Fig33.tif]

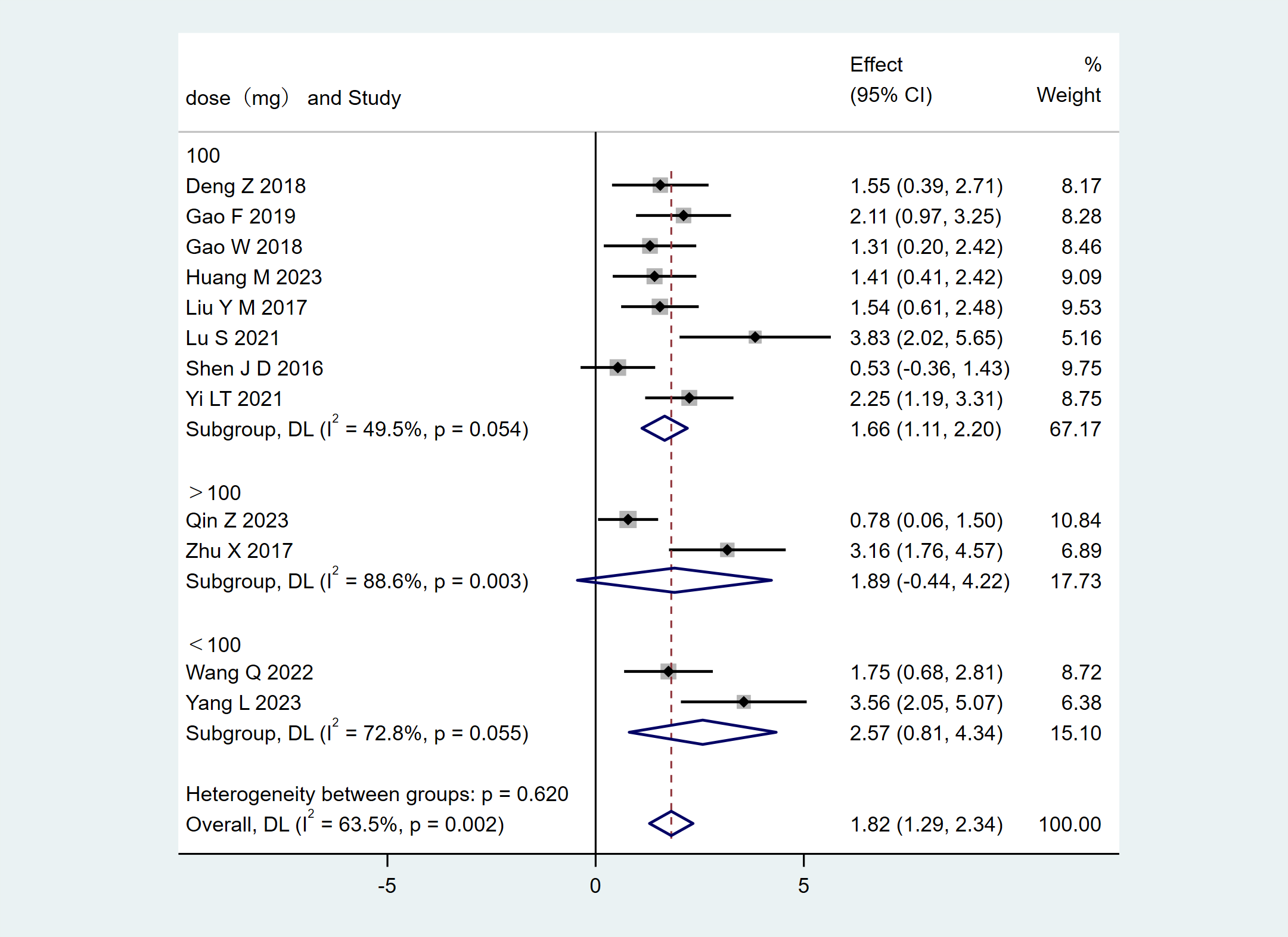

Supplement: Supplementary file 1 [file DataSheet1.zip › Supplementary Figures/Fig34.tif]

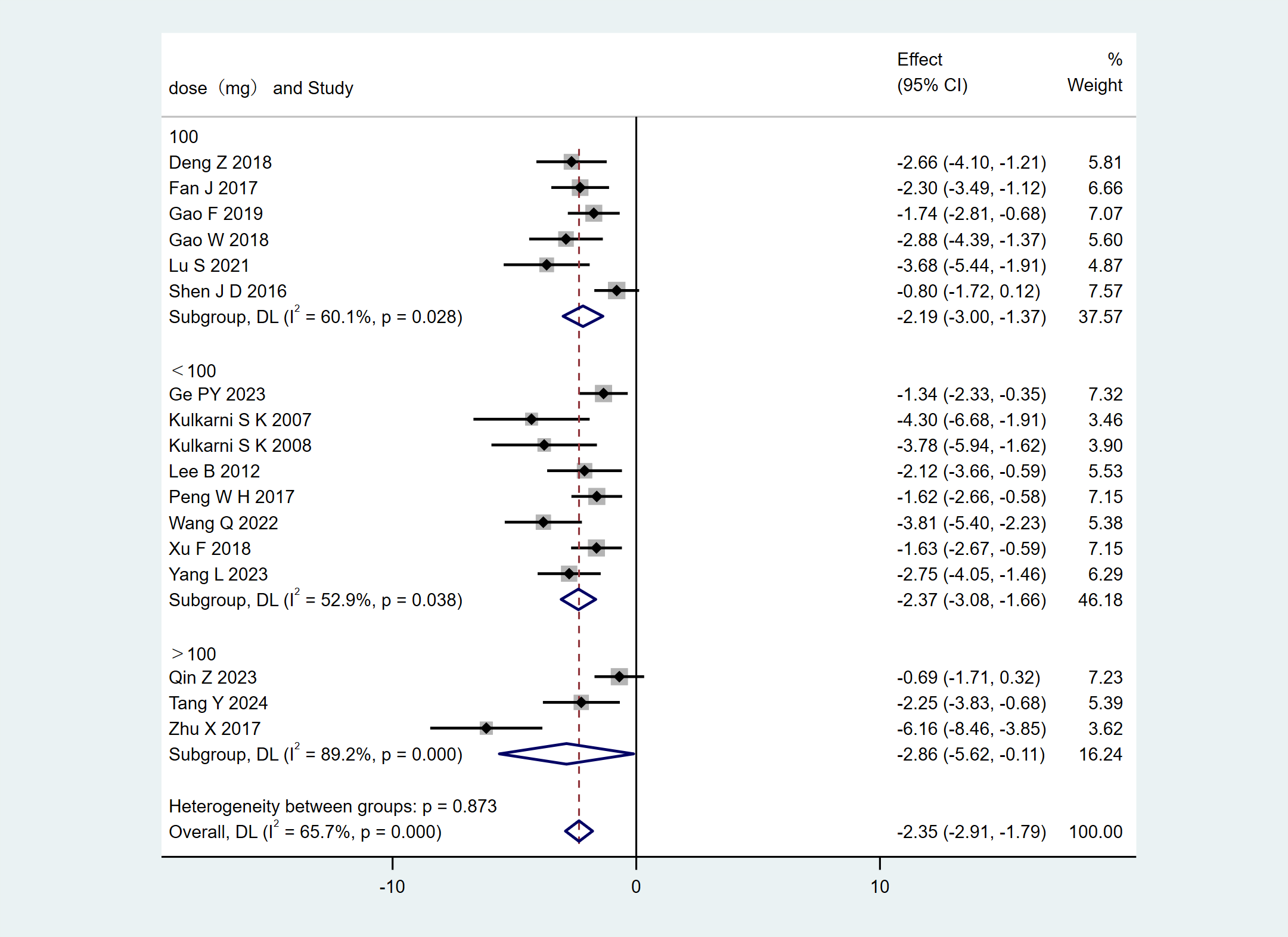

Supplement: Supplementary file 1 [file DataSheet1.zip › Supplementary Figures/Fig35.tif]

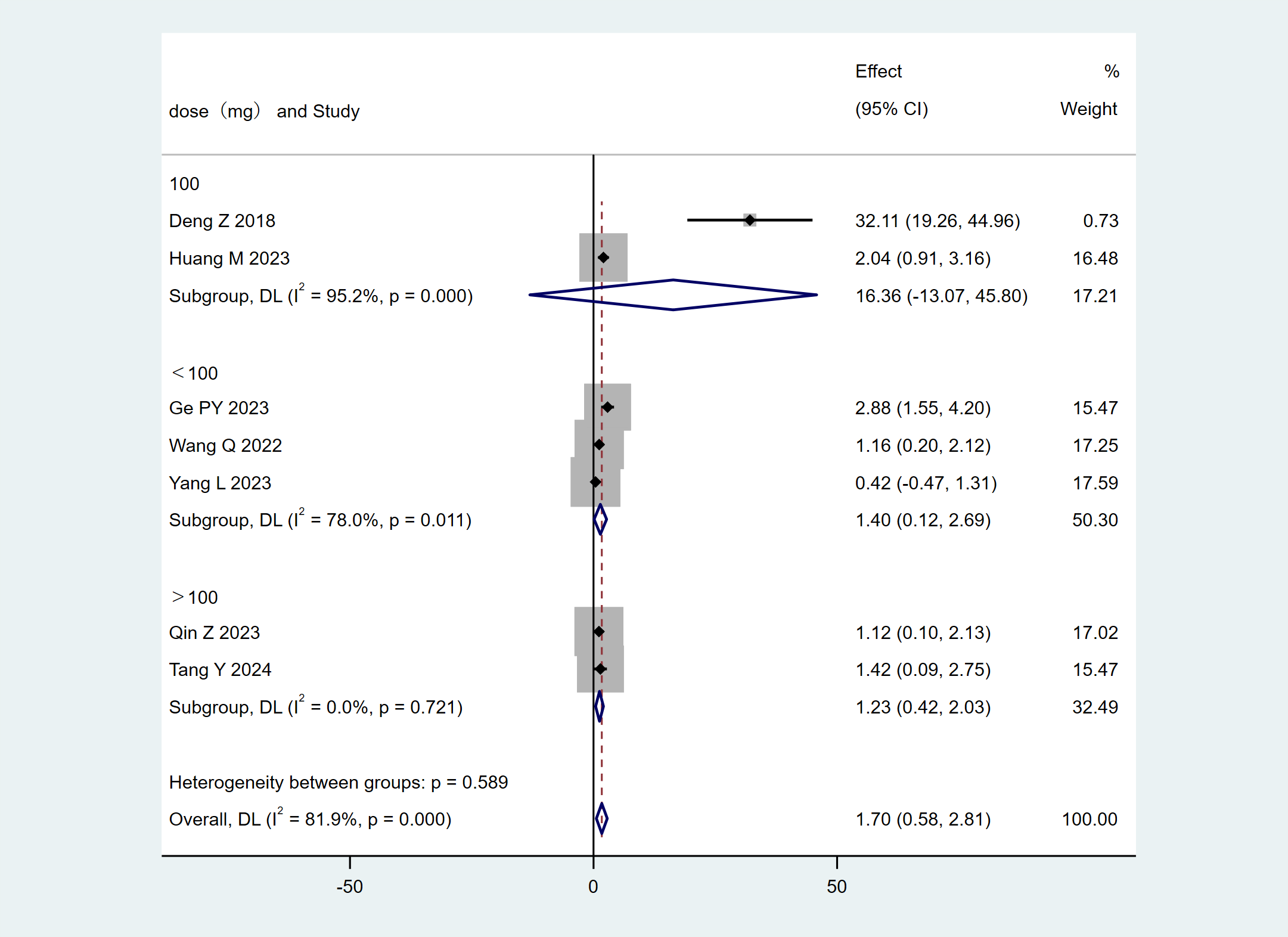

Supplement: Supplementary file 1 [file DataSheet1.zip › Supplementary Figures/Fig36.tif]

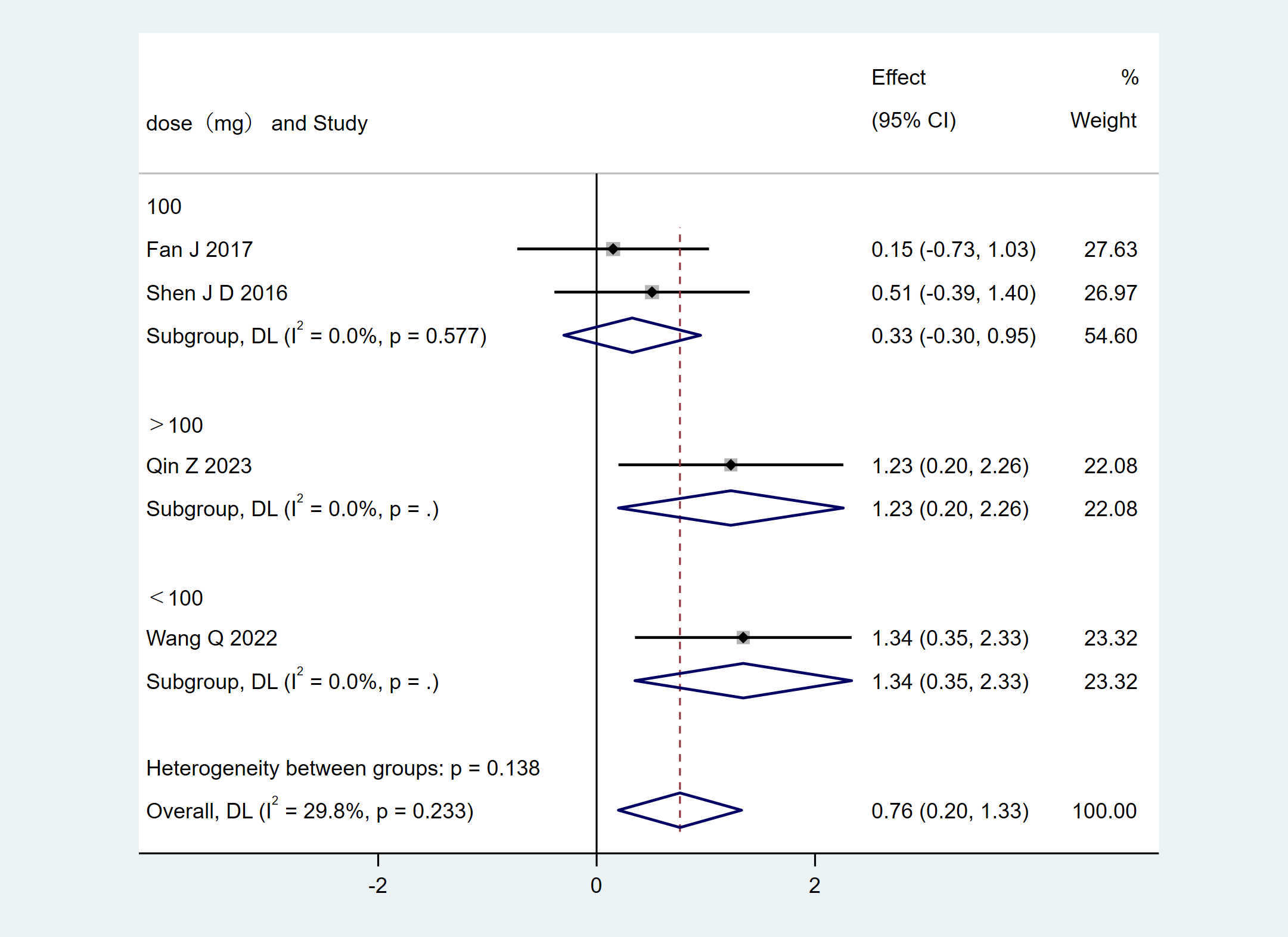

Supplement: Supplementary file 1 [file DataSheet1.zip › Supplementary Figures/Fig37.tif]

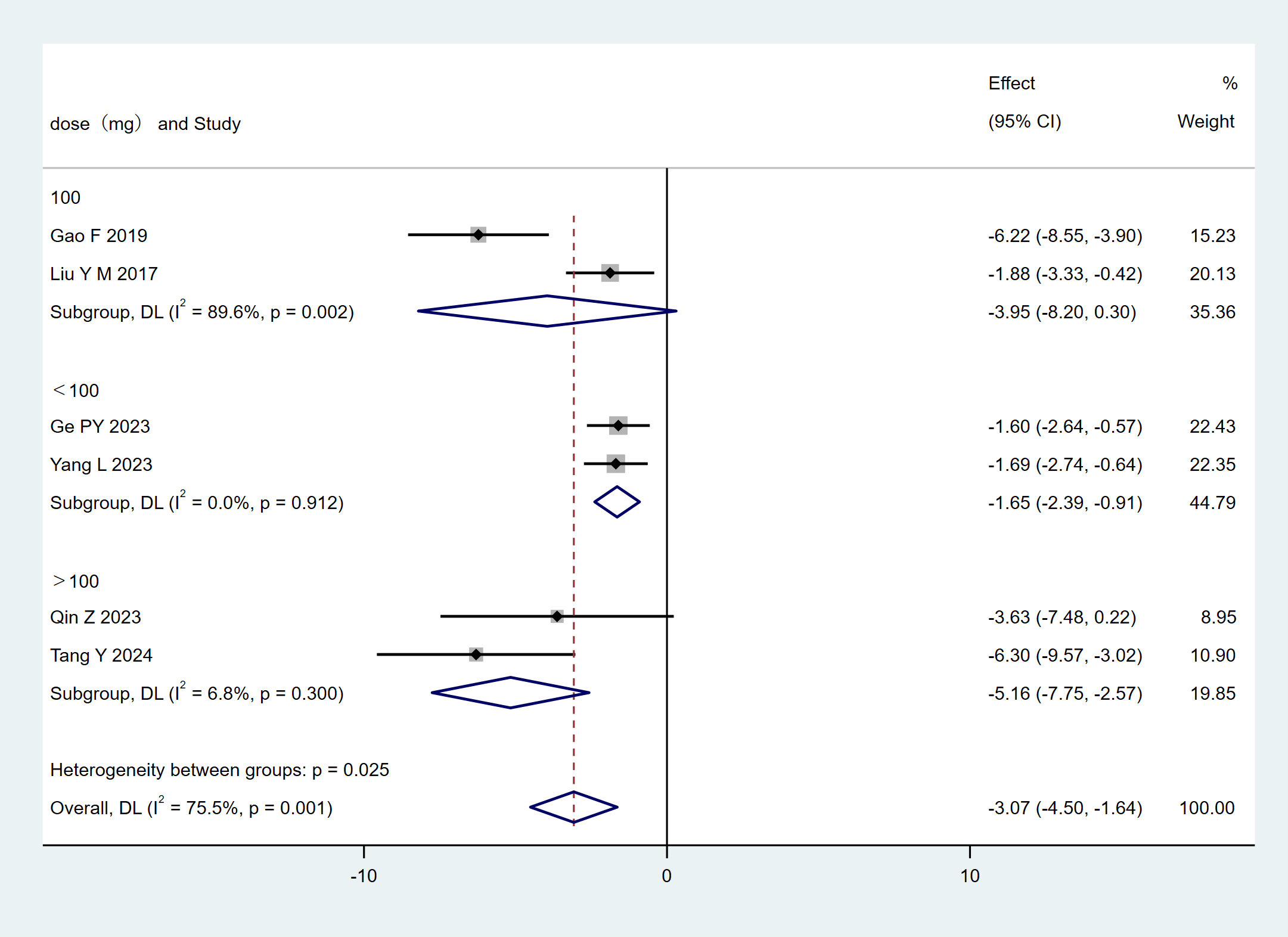

Supplement: Supplementary file 1 [file DataSheet1.zip › Supplementary Figures/Fig38.tif]

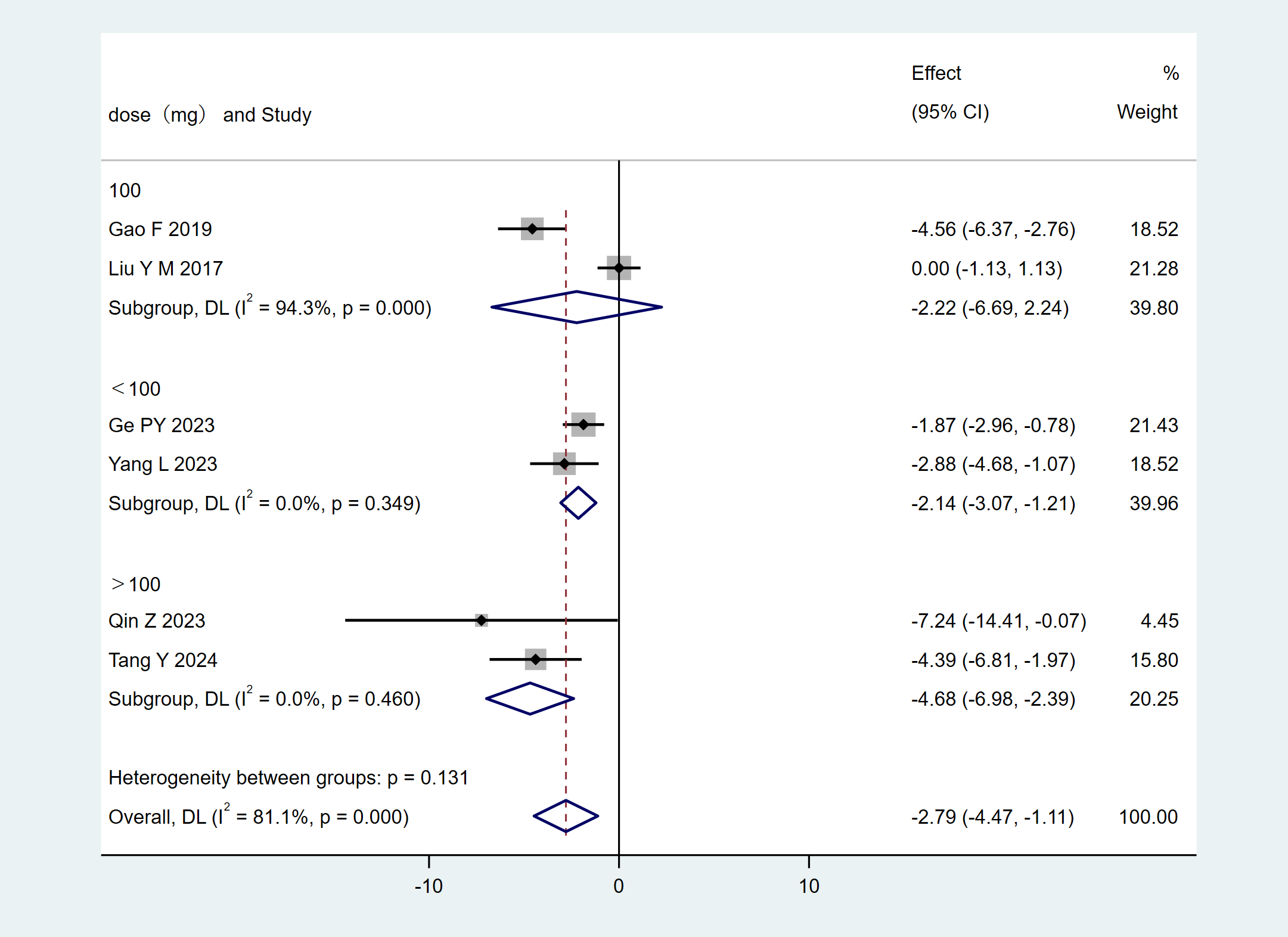

Supplement: Supplementary file 1 [file DataSheet1.zip › Supplementary Figures/Fig39.tif]

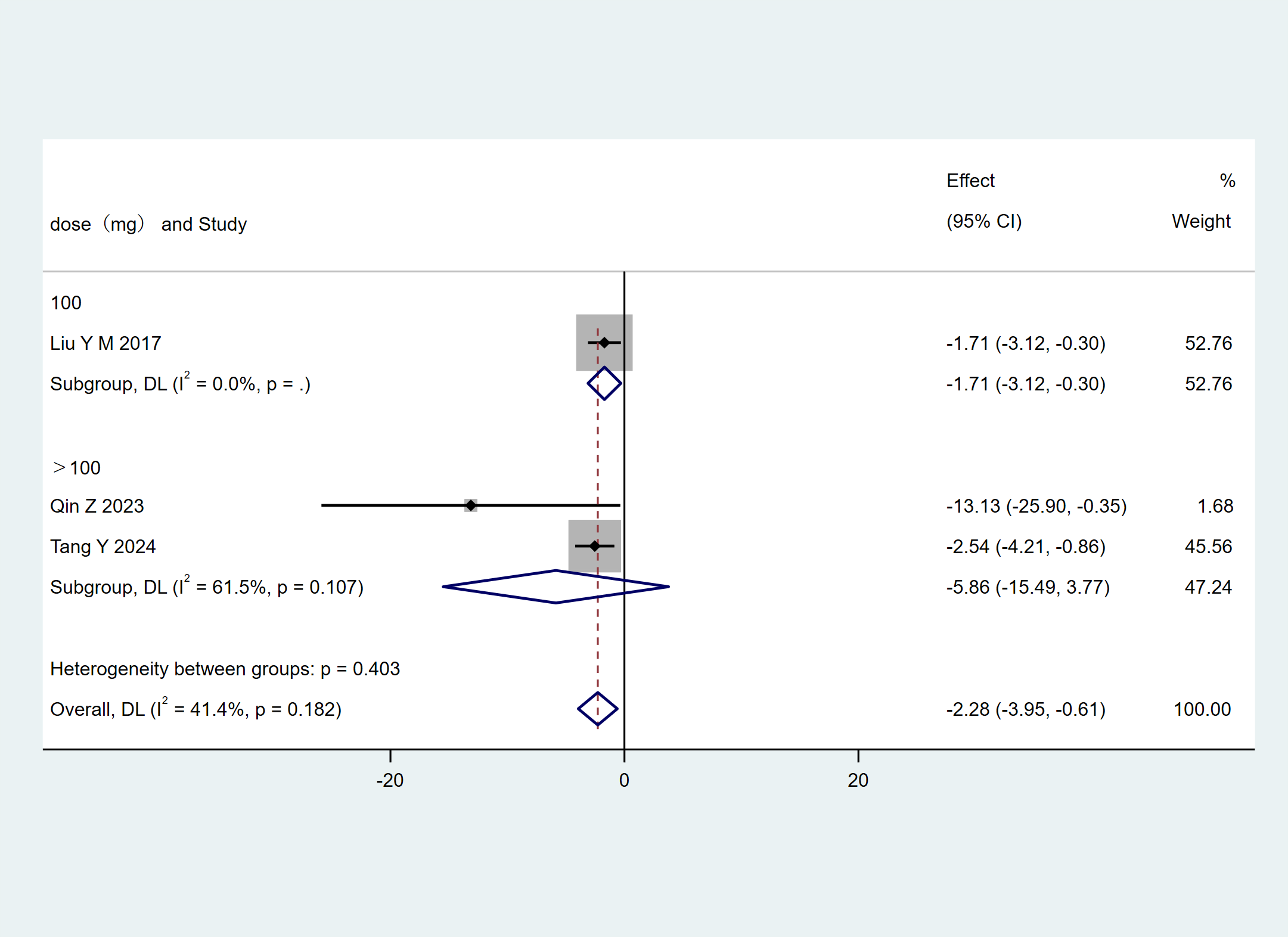

Supplement: Supplementary file 1 [file DataSheet1.zip › Supplementary Figures/Fig40.tif]

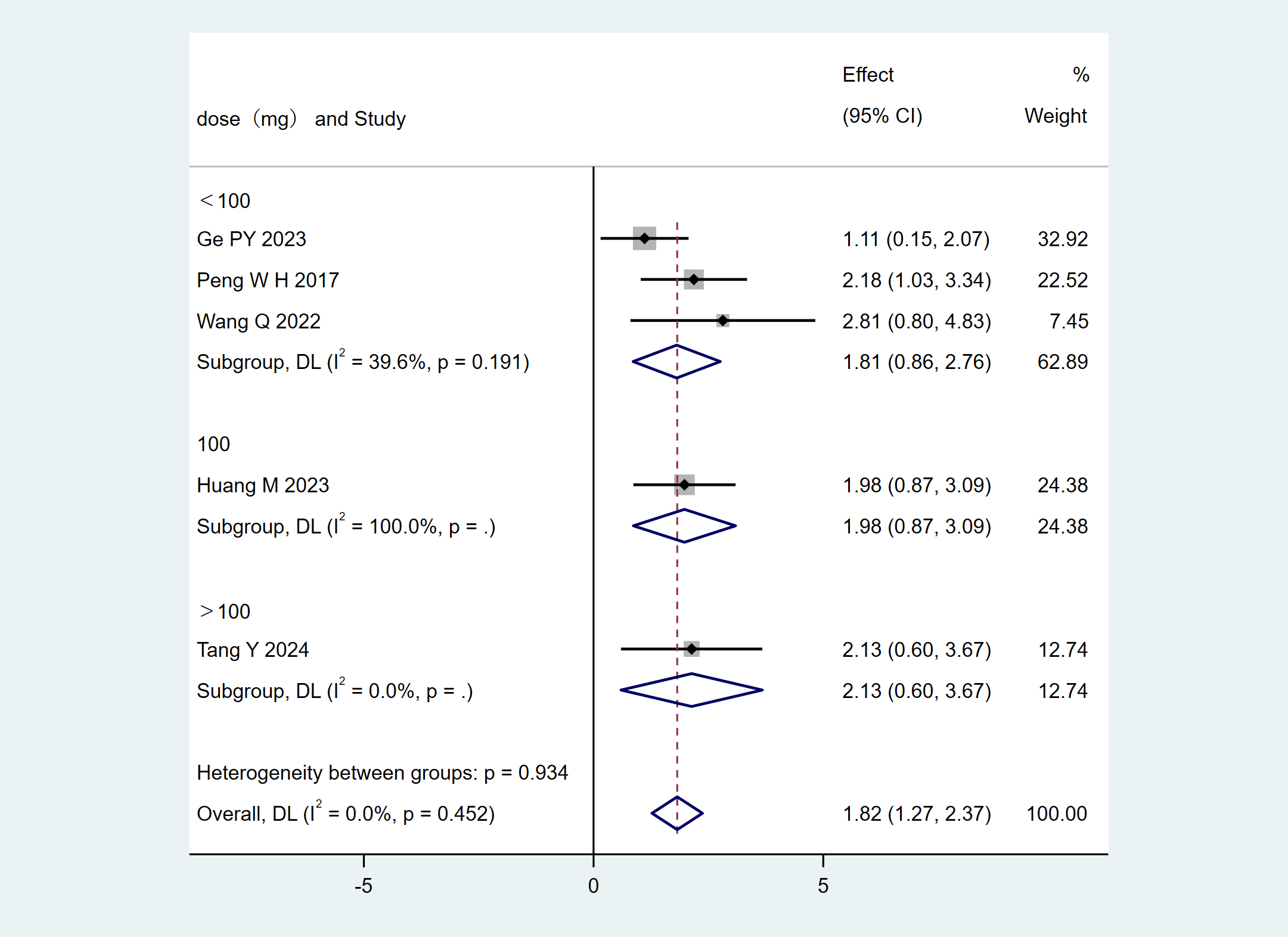

Supplement: Supplementary file 1 [file DataSheet1.zip › Supplementary Figures/Fig41.tif]

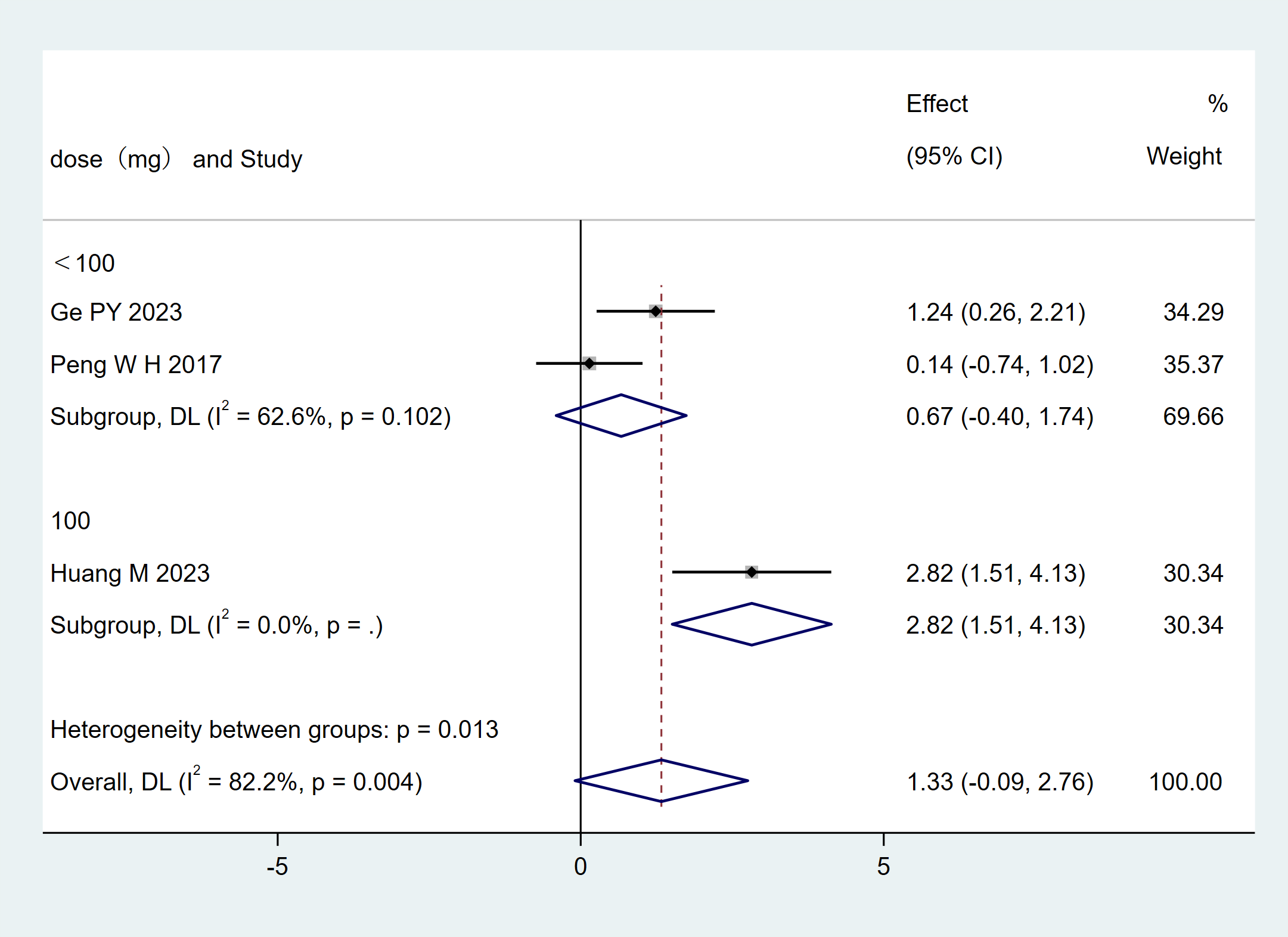

Supplement: Supplementary file 1 [file DataSheet1.zip › Supplementary Figures/Fig42.tif]

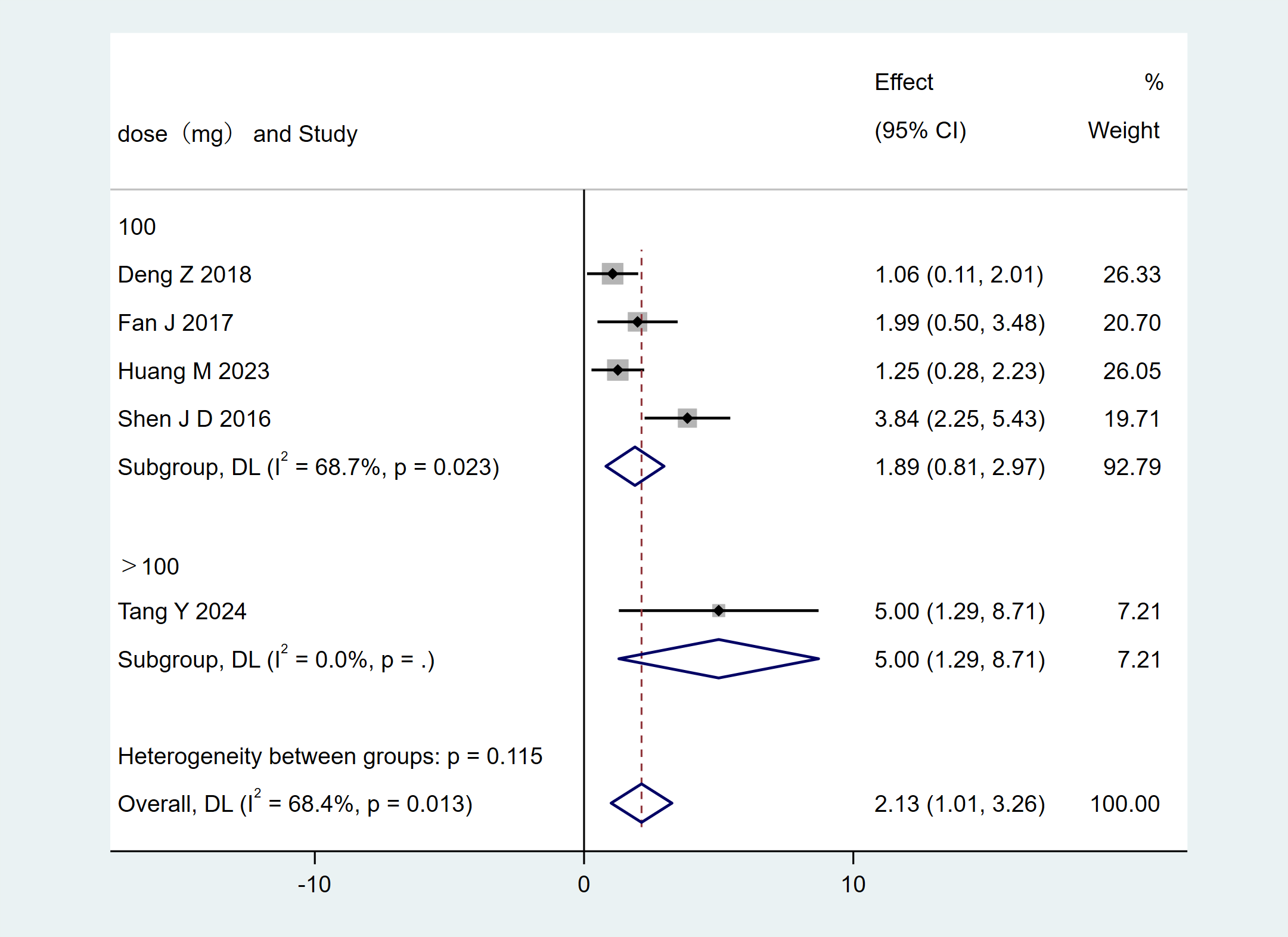

Supplement: Supplementary file 1 [file DataSheet1.zip › Supplementary Figures/Fig43.tif]

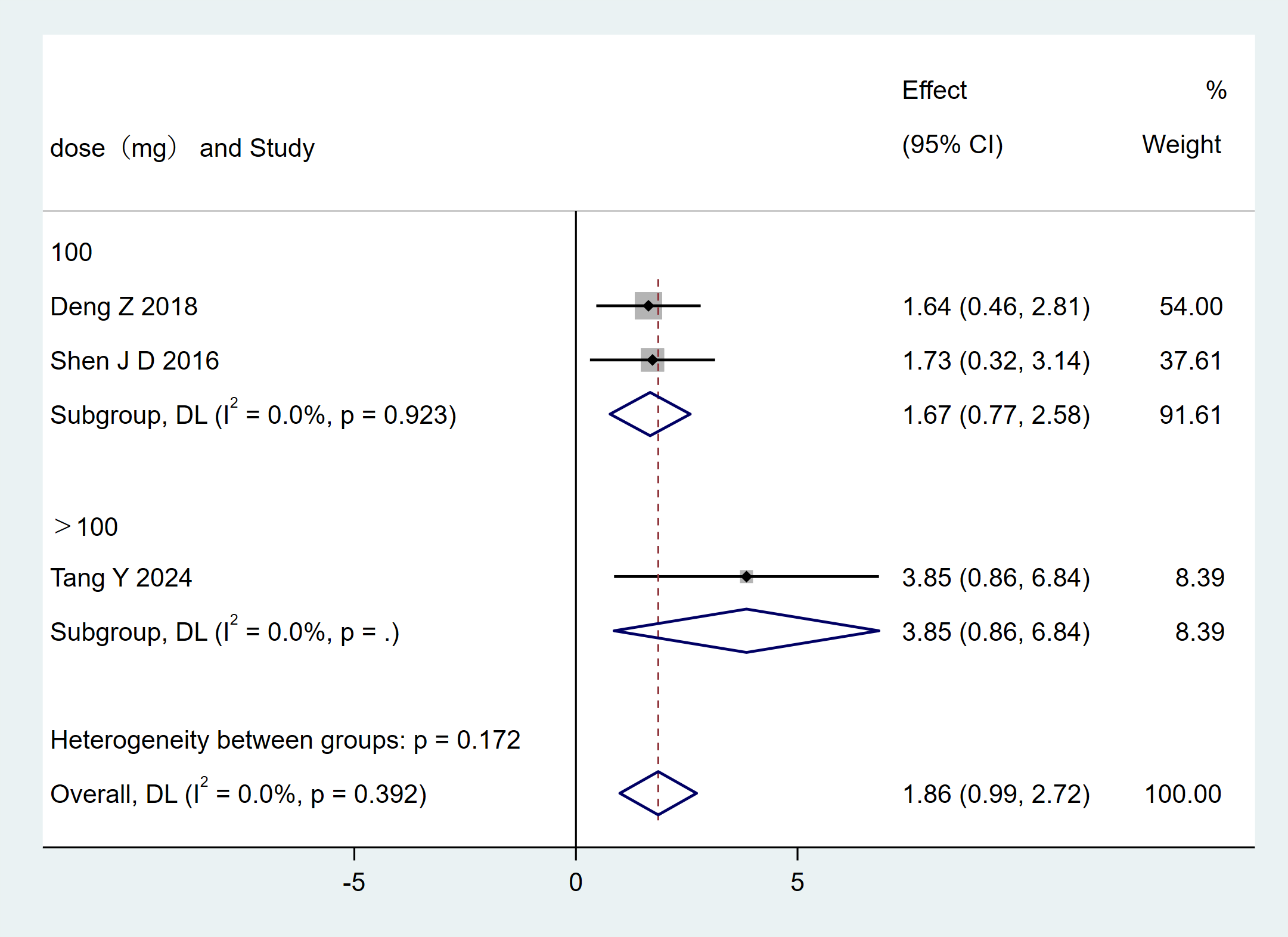

Supplement: Supplementary file 1 [file DataSheet1.zip › Supplementary Figures/Fig44.tif]

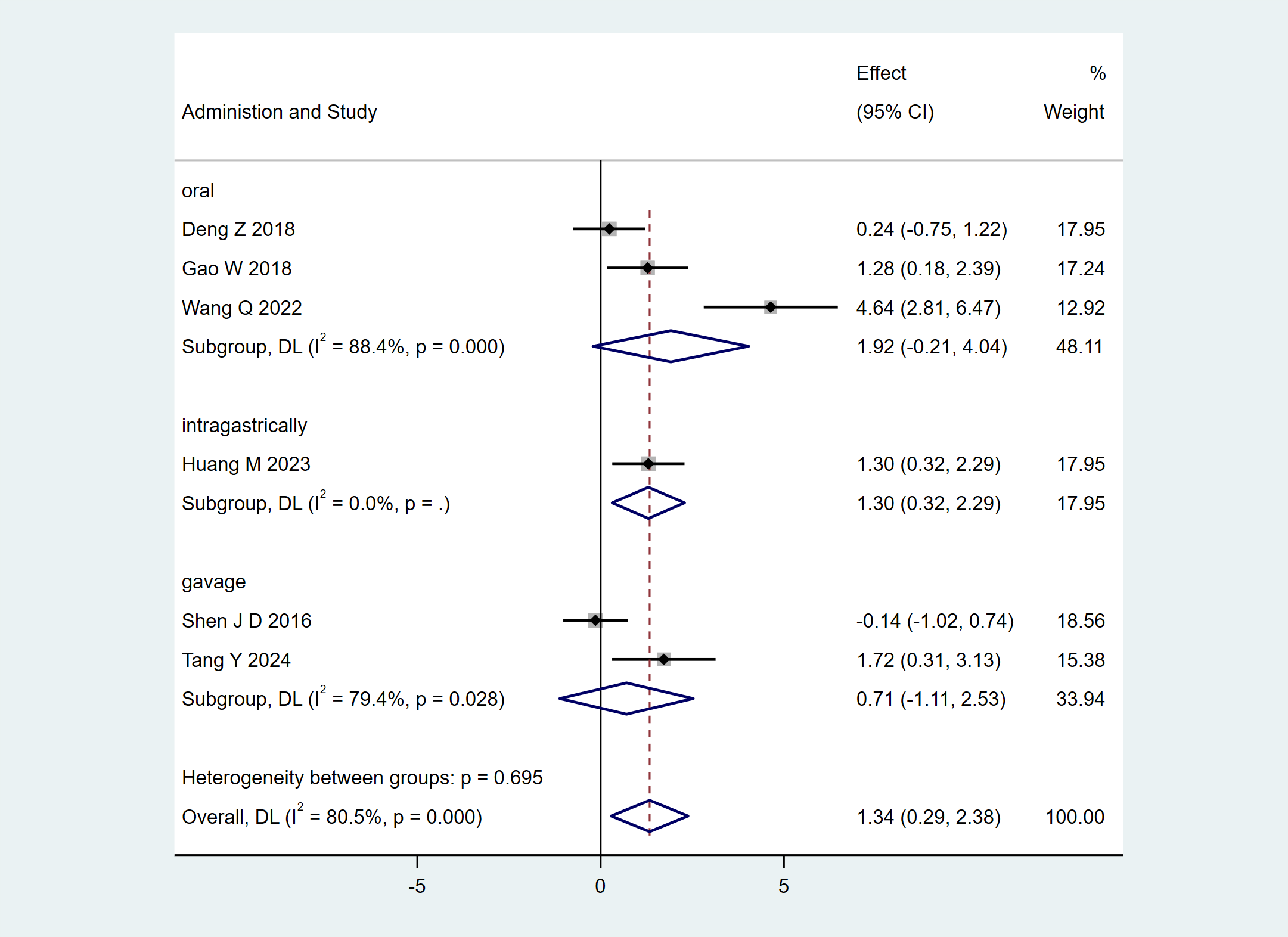

Supplement: Supplementary file 1 [file DataSheet1.zip › Supplementary Figures/Fig45.tif]

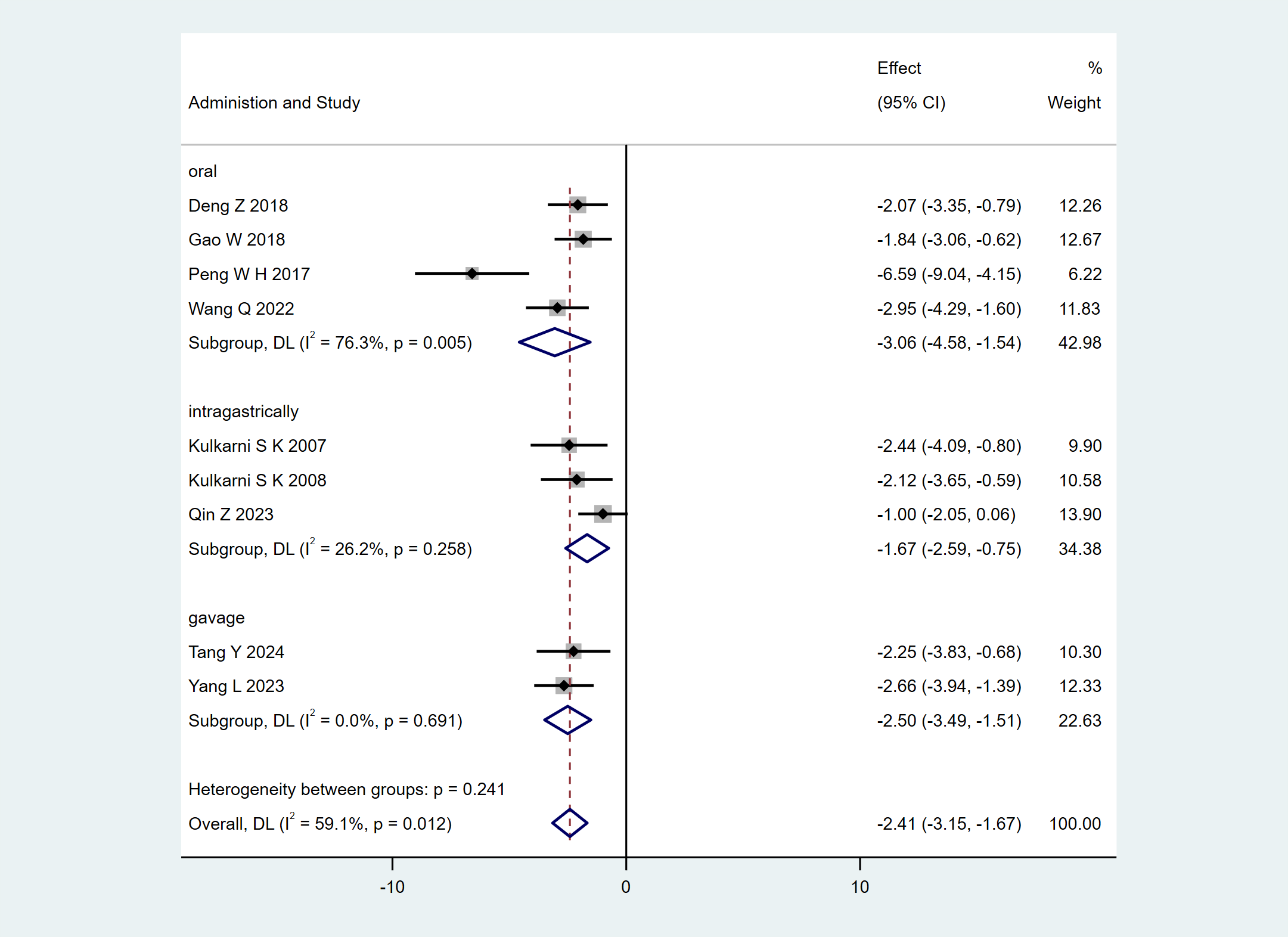

Supplement: Supplementary file 1 [file DataSheet1.zip › Supplementary Figures/Fig46.tif]

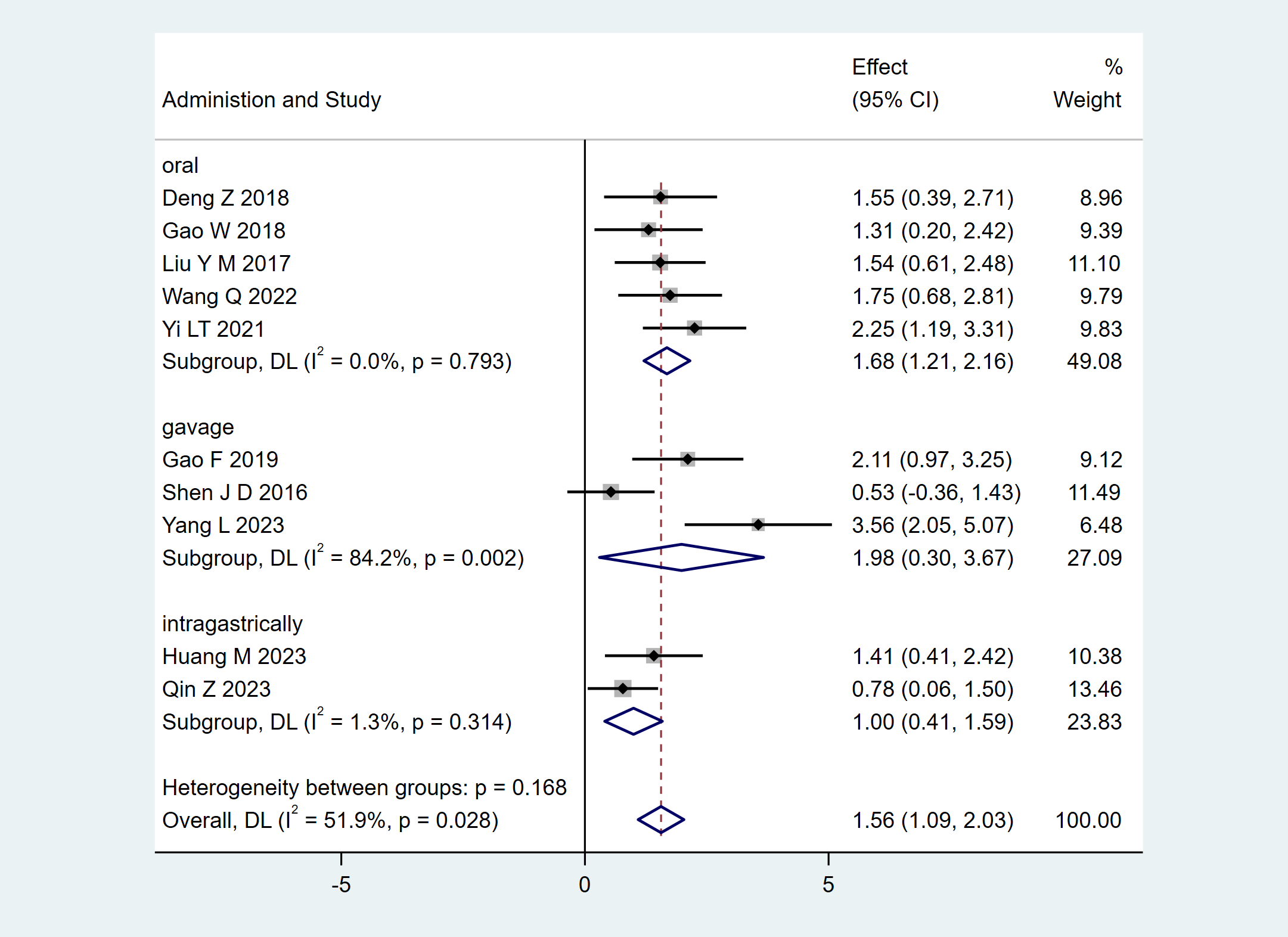

Supplement: Supplementary file 1 [file DataSheet1.zip › Supplementary Figures/Fig47.tif]

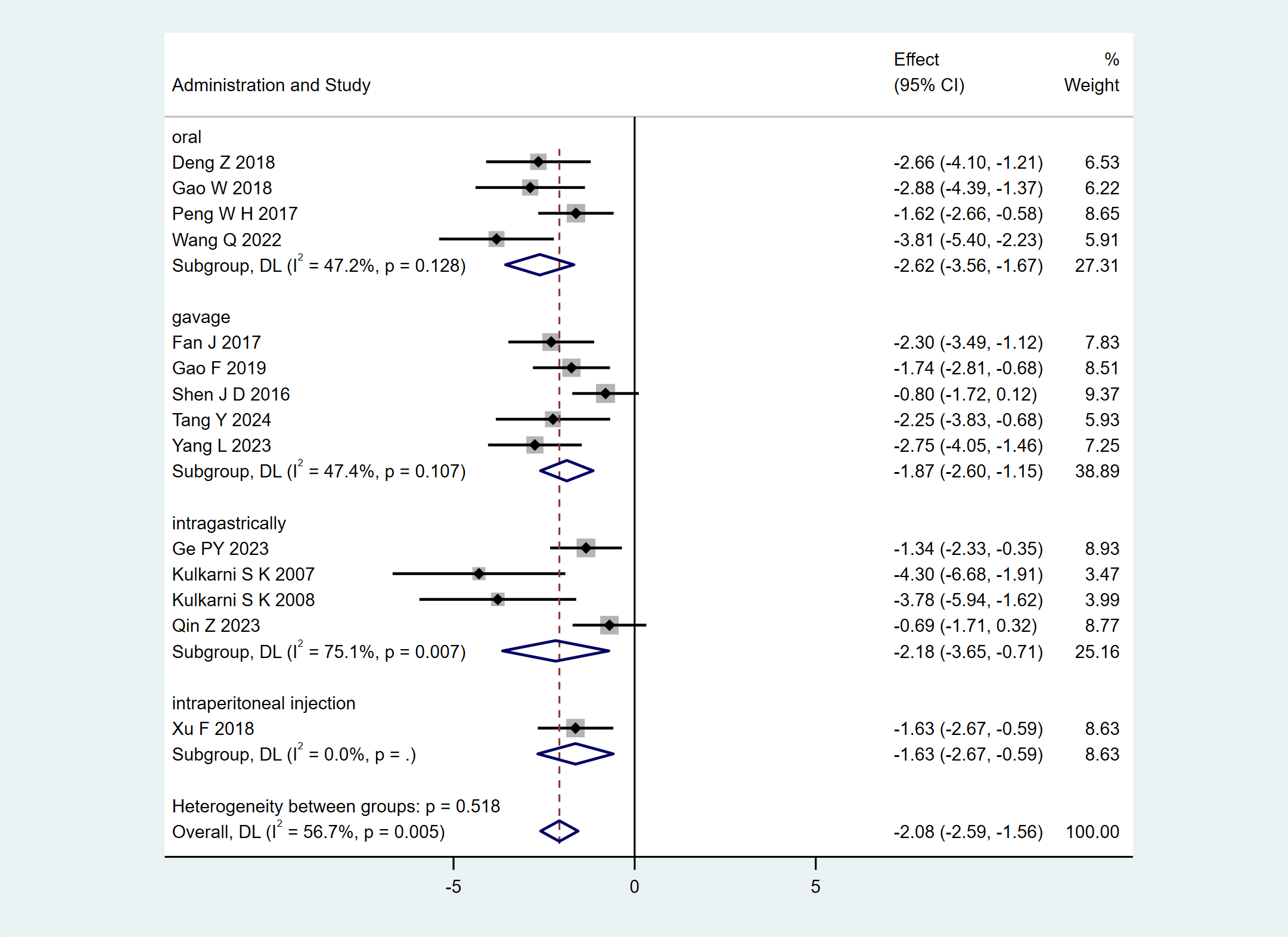

Supplement: Supplementary file 1 [file DataSheet1.zip › Supplementary Figures/Fig48.tif]

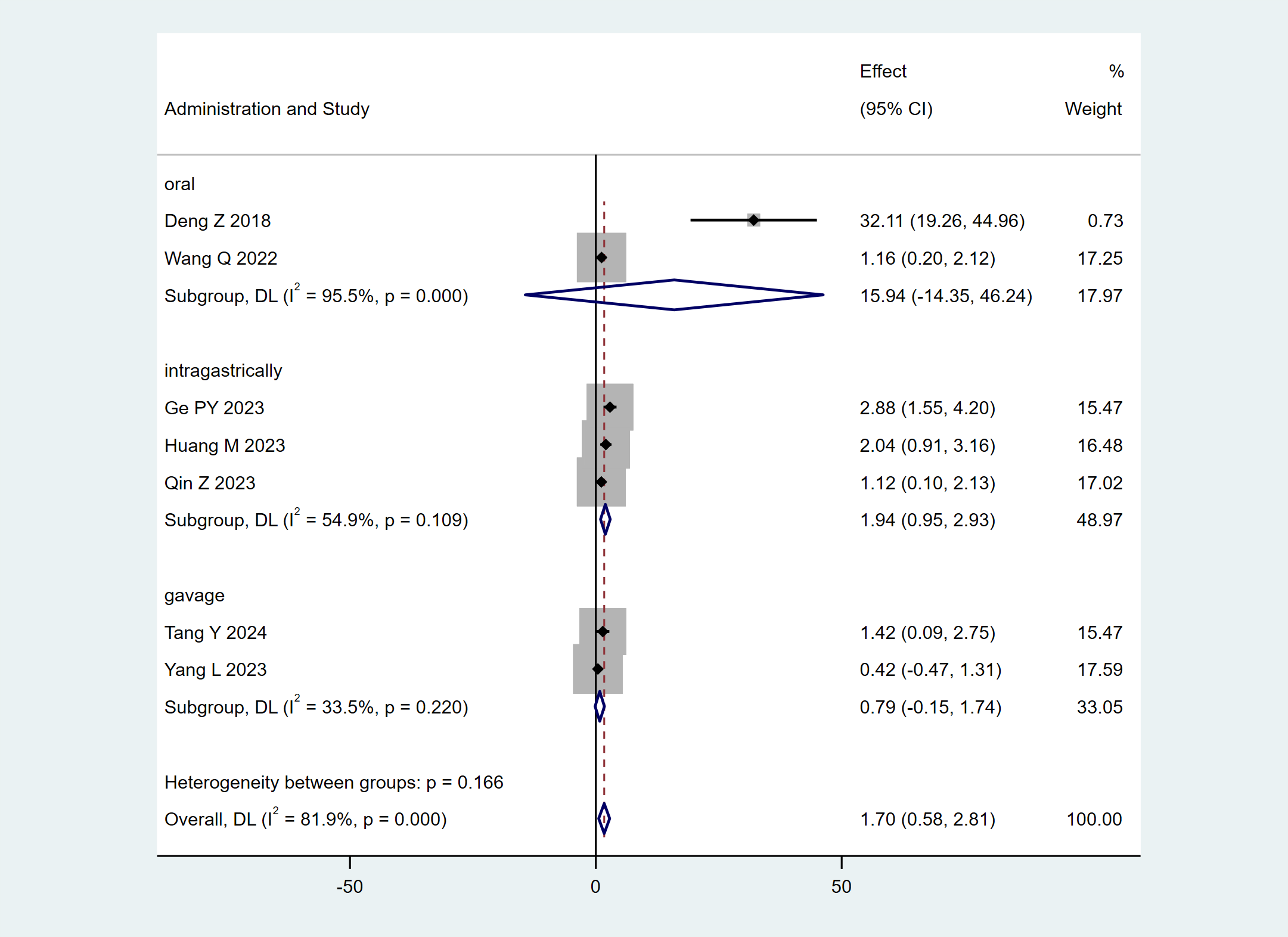

Supplement: Supplementary file 1 [file DataSheet1.zip › Supplementary Figures/Fig49.tif]

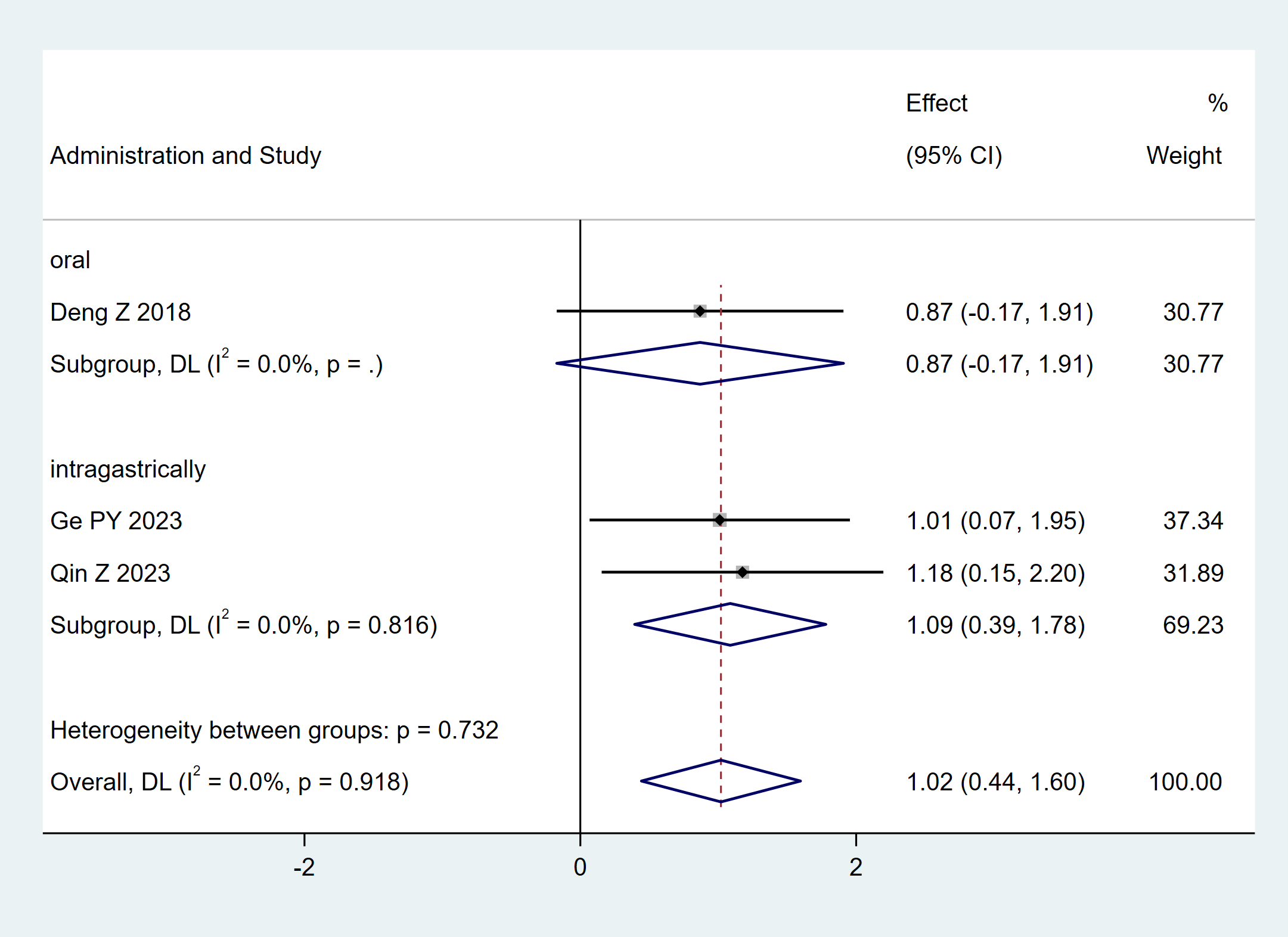

Supplement: Supplementary file 1 [file DataSheet1.zip › Supplementary Figures/Fig50.tif]

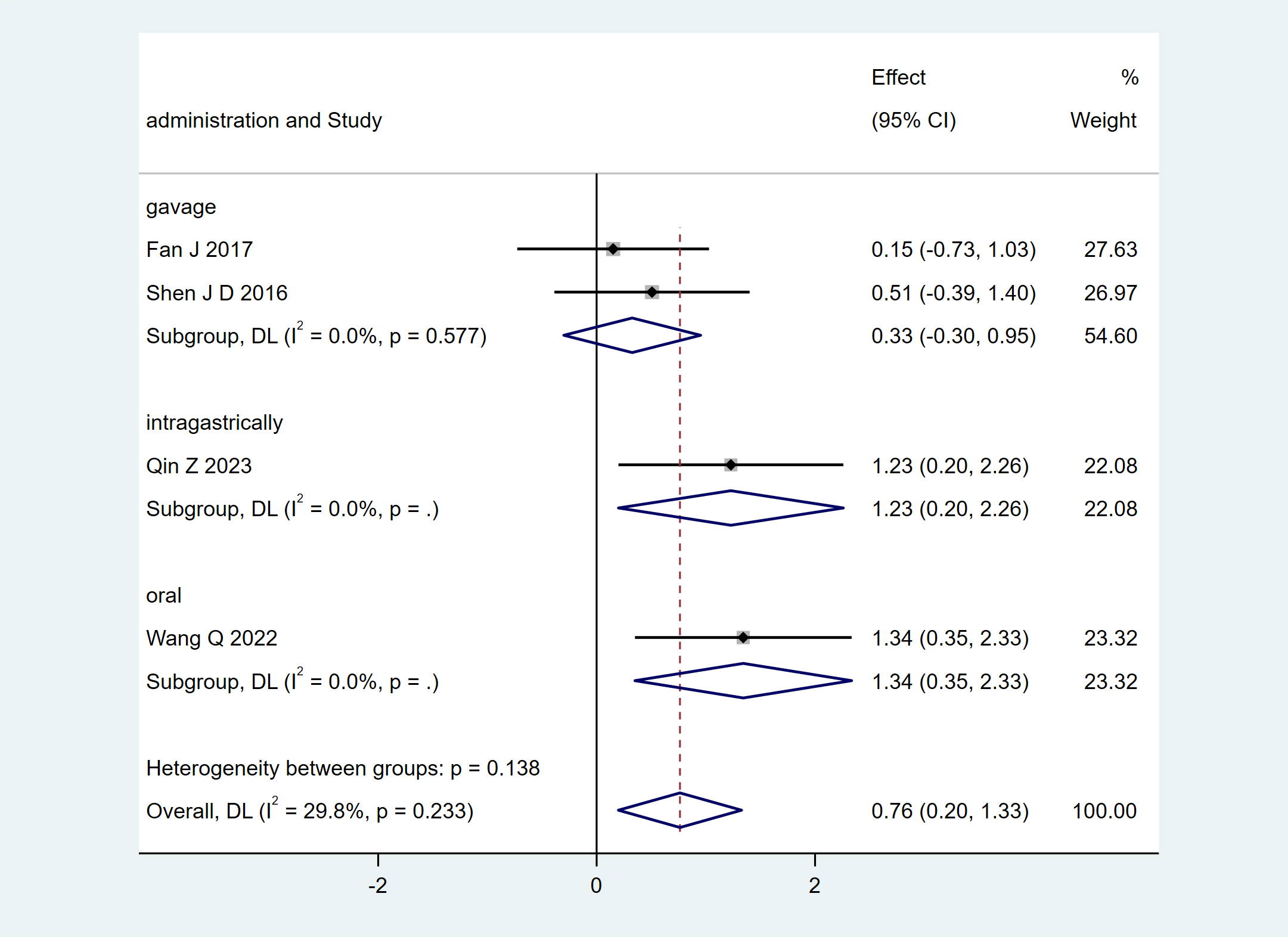

Supplement: Supplementary file 1 [file DataSheet1.zip › Supplementary Figures/Fig51.tif]

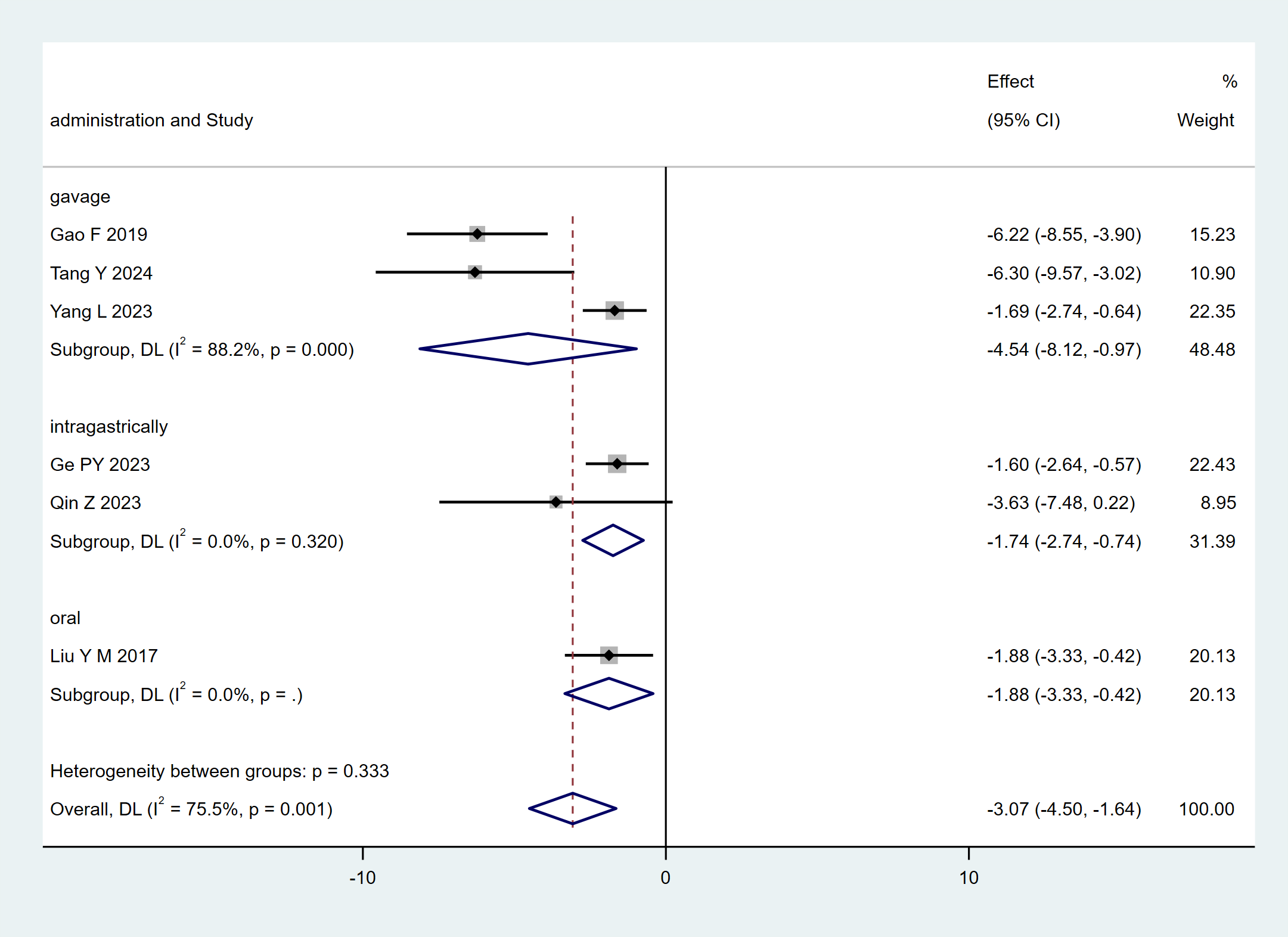

Supplement: Supplementary file 1 [file DataSheet1.zip › Supplementary Figures/Fig52.tif]

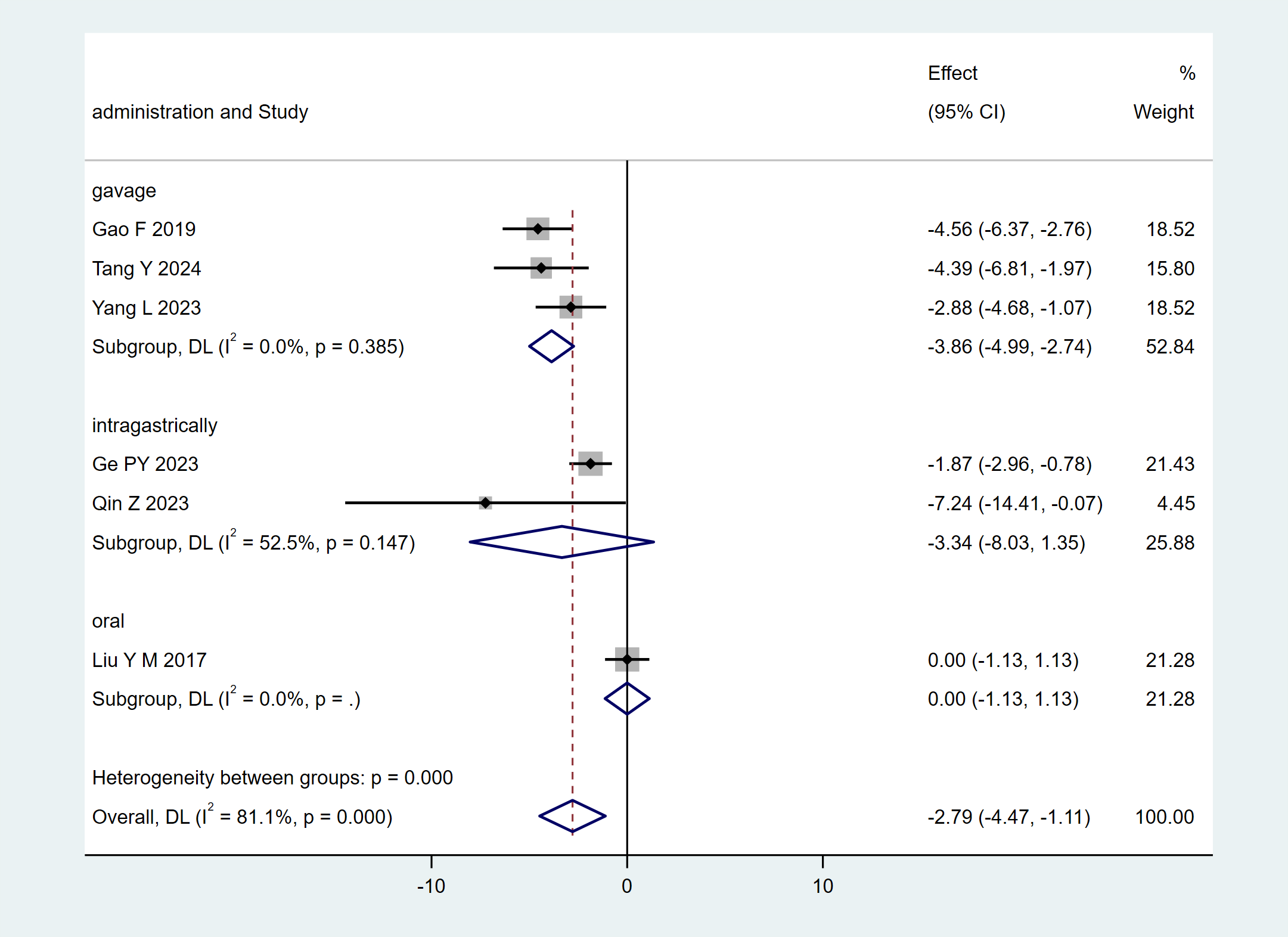

Supplement: Supplementary file 1 [file DataSheet1.zip › Supplementary Figures/Fig53.tif]

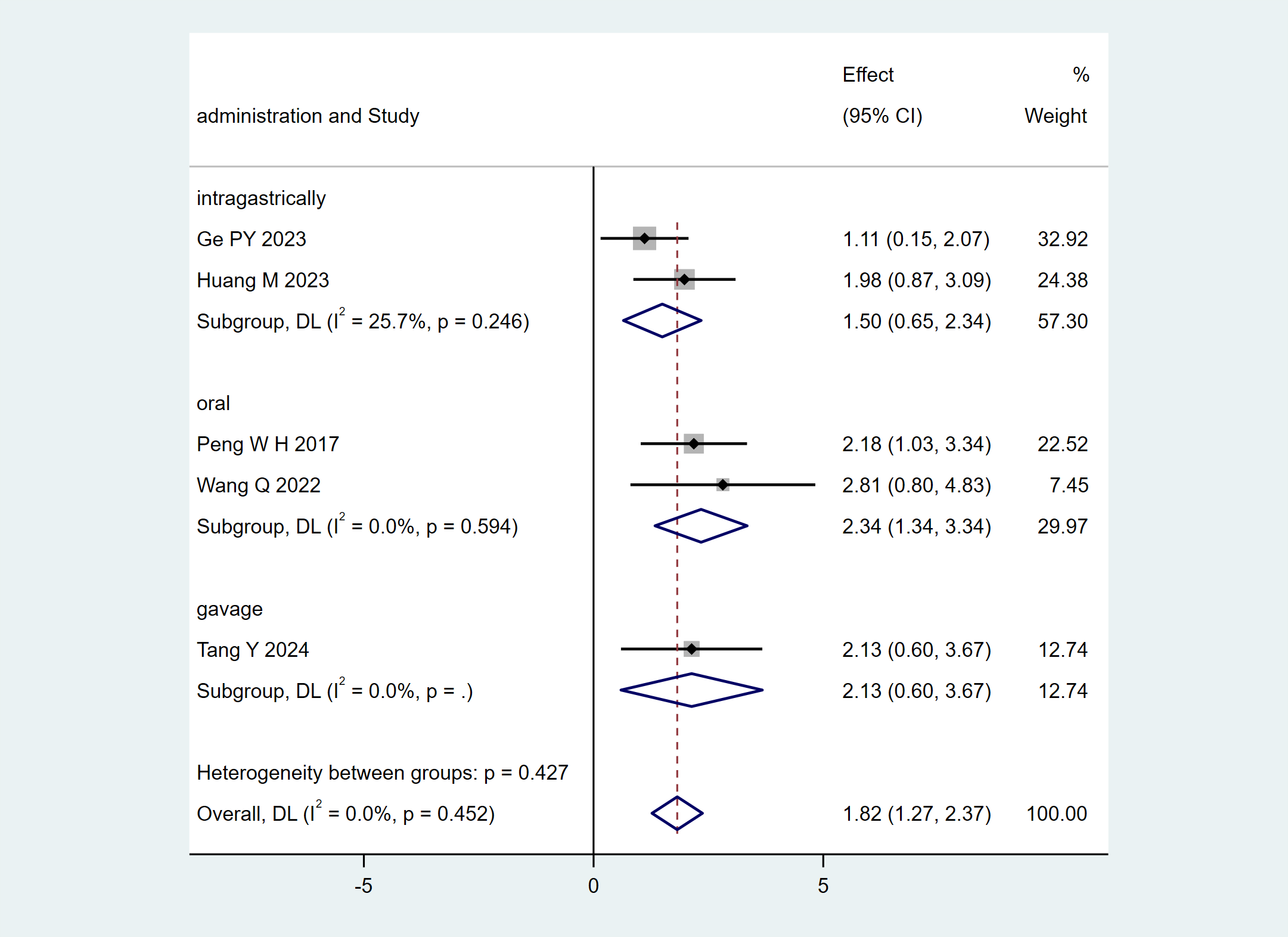

Supplement: Supplementary file 1 [file DataSheet1.zip › Supplementary Figures/Fig54.tif]

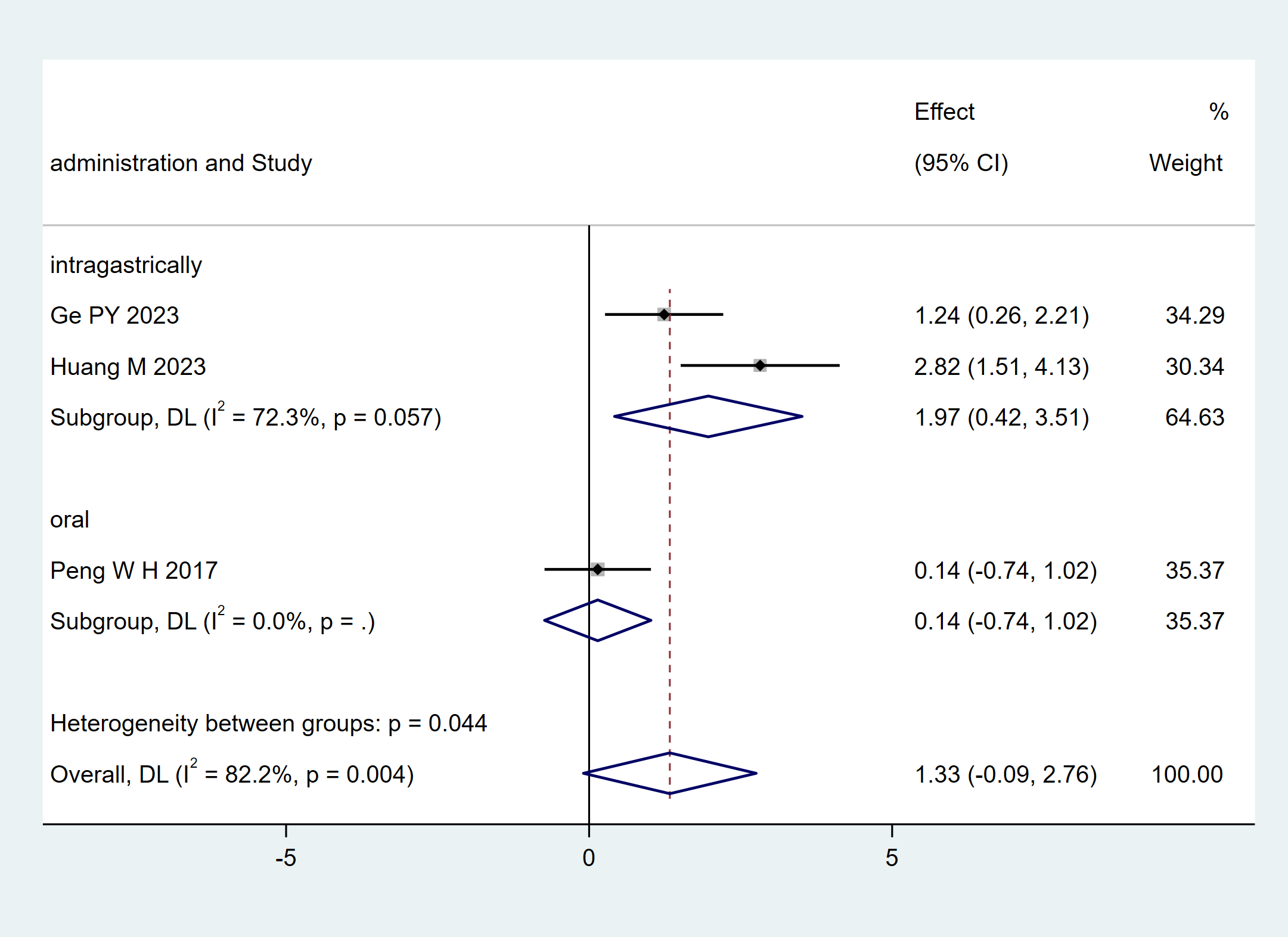

Supplement: Supplementary file 1 [file DataSheet1.zip › Supplementary Figures/Fig55.tif]

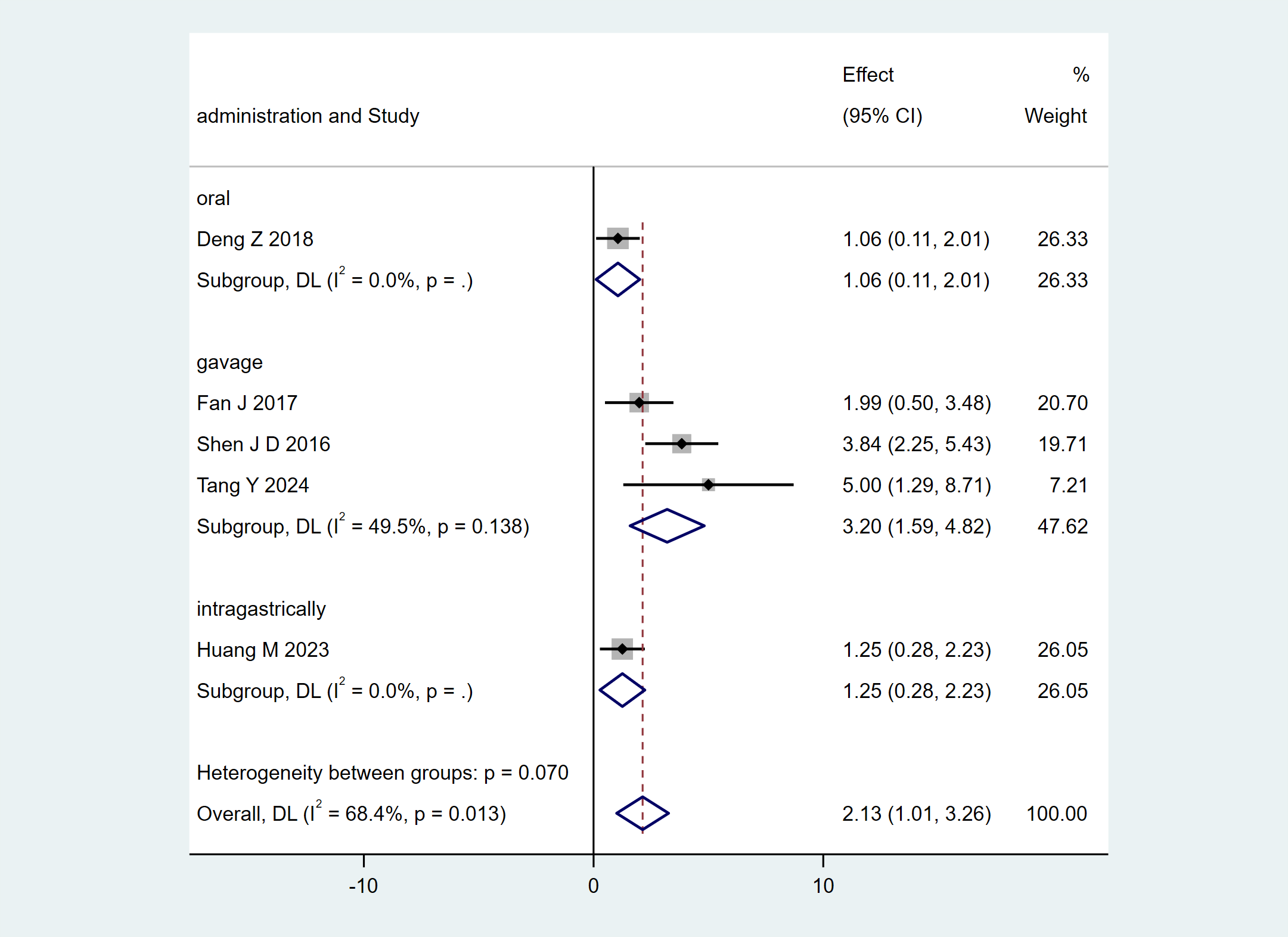

Supplement: Supplementary file 1 [file DataSheet1.zip › Supplementary Figures/Fig56.tif]

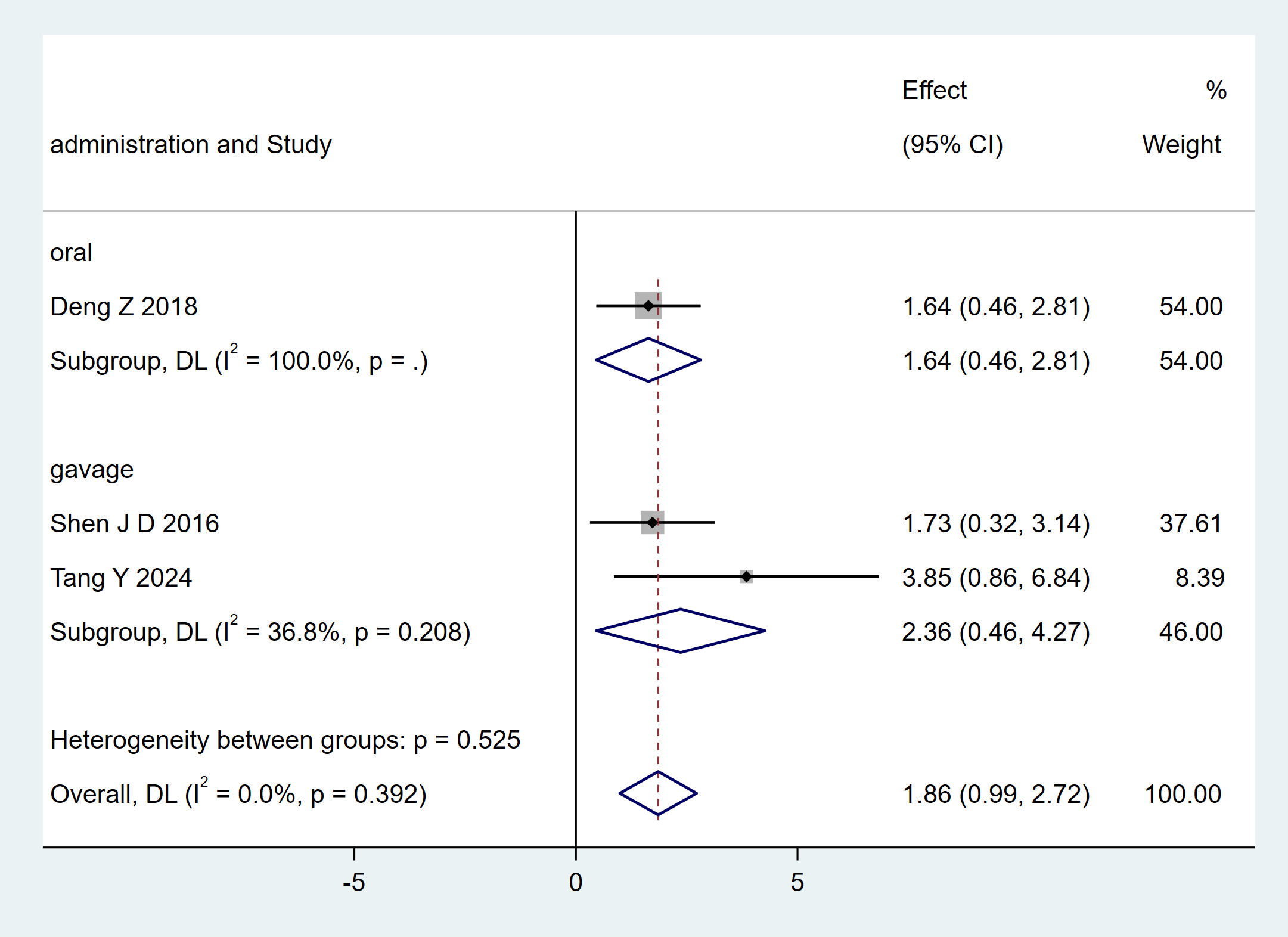

Supplement: Supplementary file 1 [file DataSheet1.zip › Supplementary Figures/Fig57.tif]
